# Coordination of Wing and Whole-Body Development at Developmental Milestones Ensures Robustness against Environmental and Physiological Perturbations

**DOI:** 10.1371/journal.pgen.1004408

**Published:** 2014-06-19

**Authors:** Marisa M. Oliveira, Alexander W. Shingleton, Christen K. Mirth

**Affiliations:** 1 Development, Evolution and the Environment Laboratory, Instituto Gulbenkian de Ciência, Oeiras, Portugal; 2 Dept. of Zoology, Michigan State University, East Lansing, Michigan, United States of America; 3 Dept. of Biology, Lake Forest College, Lake Forest, Illinois, United States of America; University of Lausanne, Switzerland

## Abstract

Development produces correctly patterned tissues under a wide range of conditions that alter the rate of development in the whole body. We propose two hypotheses through which tissue patterning could be coordinated with whole-body development to generate this robustness. Our first hypothesis states that tissue patterning is tightly coordinated with whole-body development over time. The second hypothesis is that tissue patterning aligns at developmental milestones. To distinguish between our two hypotheses, we developed a staging scheme for the wing imaginal discs of *Drosophila* larvae using the expression of canonical patterning genes, linking our scheme to three whole-body developmental events: moulting, larval wandering and pupariation. We used our scheme to explore how the progression of pattern changes when developmental time is altered either by changing temperature or by altering the timing of hormone synthesis that drives developmental progression. We found the expression pattern in the wing disc always aligned at moulting and pupariation, indicating that these key developmental events represent milestones. Between these milestones, the progression of pattern showed greater variability in response to changes in temperature and alterations in physiology. Furthermore, our data showed that discs from wandering larvae showed greater variability in patterning stage. Thus for wing disc patterning, wandering does not appear to be a developmental milestone. Our findings reveal that tissue patterning remains robust against environmental and physiological perturbations by aligning at developmental milestones. Furthermore, our work provides an important glimpse into how the development of individual tissues is coordinated with the body as a whole.

## Author Summary

Between distantly related species, development converges at common morphological and genetic stages, called developmental milestones, to ensure the establishment of a basic body plan. Beyond these milestones greater variability in developmental processes builds species-specific form. We reasoned that developmental milestones might also act within a species to achieve robustness against environmental or physiological perturbation. To address this, we first developed a staging scheme for the progression of pattern in the wing disc across developmental time. We then explored how perturbing environmental or physiological stimuli known to alter the rate of development affected the progression of pattern in the wing disc. We found two developmental milestones, the moult to the third instar and pupariation, where wing disc patterning aligned with the development of the whole body. This suggests that robustness against environmental and physiological conditions is achieved by coordinating tissue with whole-body development at developmental milestones.

## Introduction

Organisms require robust developmental processes to guarantee that developing tissues pattern correctly in the face of a wide range of environmental and physiological perturbations [1], [2]. A developmental process can be considered robust if variation in this process is uncorrelated with variation in genetic, environmental or physiological conditions [3]. To achieve robustness, the developmental processes that generate individual organs must, at some level, be integrated across the whole body to ensure that a correctly patterned and proportioned adult is produced at the end of development. It is therefore thought that the progression of gene expression that occurs in tissues as they pattern needs to be somehow integrated with the systemic hormone levels that trigger transitions between developmental stages (hereafter termed developmental events) across the whole body [4], [5]. The timing of these developmental events changes with environmental and physiological conditions but how this affects tissue development is not fully understood.

**Figure 1:**
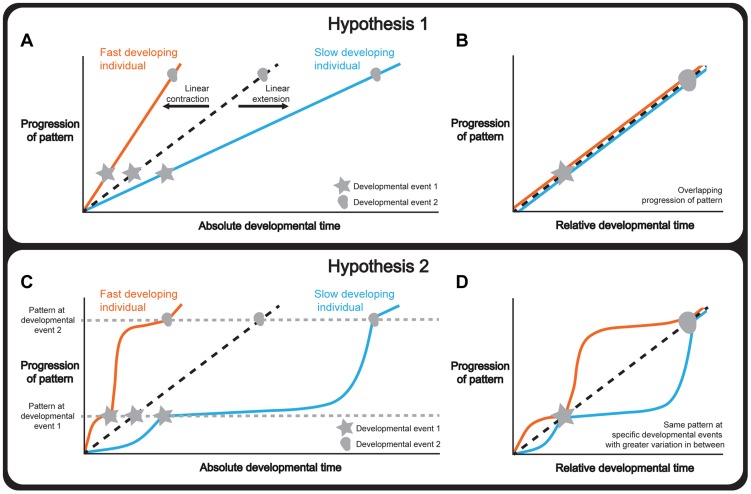
Hypotheses to explain how organ and whole-body development are coordinated. (a-b) Hypothesis 1: Whole-body development and individual tissue patterning are tightly coordinated throughout development. Changing the length of time required for development extends or contracts the progression of pattern in a linear manner (a) and, consequently, normalizing the data to a developmental endpoint, referred to as relative developmental time, produces overlapping progressions of pattern (b). (c-d) Hypothesis 2: Whole-body development and individual tissue patterning are coordinated only at key physiological transitions. Changing the length of time required for development alters the relationship between tissue patterning and developmental time non-linearly and patterning converges only at these transitions (c). Consequently, in relative developmental time, the progression of pattern overlaps at these events and shows greater variability in the intervals between them (d). Note that the curve of the two lines between the developmental events is illustrative showing two ways the curves could differ under altered developmental conditions. Tissue patterning of a reference/condition is represented in black dashed lines; fast developers are represented in orange and slow developers are shown in blue. Stars and circles symbolize developmental events 1 and 2.

There are several hypotheses to explain how tissue patterning is integrated with whole-body development under different environmental and physiological conditions. One hypothesis is that tissue patterning and whole-body development progress synchronously, so that the rate of the former matches the rate of the latter. If this were the case, a change in the duration of development would extend or contract the progression of patterning in a linear manner (Figure 1a). Consequently, normalizing the progression of pattern to a developmental endpoint, that is using relative rather than absolute developmental time, would produce the same progression of patterning independent of the duration of development (Figure 1b).

Alternatively, tissue patterning may only be coordinated with whole-body development at key developmental events (Figure 1c), for example moulting in holometabolous insects, or the onset of puberty in humans. Although not all developmental events act to coordinate, those that do are often referred to as developmental milestones [6]. Thus if the duration of development varies, the progression of patterning would nonetheless converge at these milestones while showing greater variability between them. Consequently, normalizing the progression of pattern to relative developmental time would produce patterns that overlapped only at developmental milestones (Figure 1d). This would essentially mean that if patterning were to drift in rate with respect to whole-body development, developmental milestones would ensure that the rate of patterning would decelerate or accelerate to achieve the correct stage by the onset of the milestone.

Problematically, it has been difficult to test these alternative hypotheses because, while the process of patterning has been described in exquisite detail in a variety of tissues, the dynamics of patterning is rarely tied to organismal age or whole-body physiology. Several authors have explored how genetic background contributes to the robustness of development (see examples [7], [8]). Their approaches have focussed on the endpoints of development and on changes in the sequences of specific patterning cascades. Furthermore, studies in organisms ranging from insects to nematodes to vertebrates have explored the progression of gene expression in relation to embryonic stage to identify developmental milestones, called phylotypic stages, where gene expression converges upon an embryonic stage common across species [6], [9]–[12]. Such developmental milestones are thought to constrain development like an hourglass, as development across species varies more both before and after the milestones [6], [9], [10], [12]. However, these studies do not address how environmental/physiological conditions affect the progression and sequence of pattern, and how this is coordinated with whole-body development within a species. We therefore took advantage of the extensive knowledge of tissue patterning and whole body physiology of the fruit fly, *Drosophila melanogaster*, to elucidate the extent to which tissue patterning is coupled with whole-body development.

In *Drosophila*, the juvenile period comprises three larval moults. This is followed by a wandering stage where larvae leave the food and search for a pupariation site. Larval development ends with pupariation, whereupon the fly metamorphoses into its adult form. These events provide useful markers of whole-body development. Each of these developmental events (moulting, wandering and pupariation) is regulated by pulses in the titre of the steroid hormone ecdysone [13], synthesized by the prothoracic gland.

Most of the adult tissues of *Drosophila* arise from pouches of cells that grow and pattern within the body of the developing larvae, the imaginal discs [14]–[17]. Pulses of ecdysone have also been shown to regulate some stages of imaginal disc development. Early in the third larval instar a pulse of ecdysone controls the expression of three patterning gene products, Cut (Ct), Senseless (Sens) and Wingless (Wg), in response to nutrition [17]. After pupariation, ecdysone regulates Sens expression to control the differentiation of sensory organs in the wing [15], [16]. Thus, these pulses of ecdysone have been interpreted to be checkpoints that coordinate the patterning and development of tissues with whole-body developmental events [5], [18]. Nevertheless, it remains to be determined if this coordination between tissues and the whole body is necessary and happens at all developmental events, or only at specific developmental milestones.

The rate of developmental progression and the timing of these developmental events can be altered both environmentally and by genetically manipulating the timing of ecdysone synthesis. For example, *Drosophila* larvae raised at lower temperatures take longer to eclose as adults [19]–[23] while larvae reared at higher temperatures eclose more quickly [20], [22]. Similarly, altering the timing of ecdysone synthesis, by suppressing or activating insulin signalling in the prothoracic gland, also changes developmental timing and retards or accelerates eclosion [17], [24], [25].

To test the extent to which whole-body development and the progression of pattern in individual tissues are coordinated, we first generated a staging scheme to describe how patterning progresses over time in the wing imaginal discs of third instar larvae. This staging scheme was based on the changes in expression pattern of key patterning genes. We then altered developmental rate either environmentally, by using temperature manipulations, or physiologically, by altering the timing of ecdysone synthesis. We compared the progression of patterning, as determined by our staging scheme, in larvae that differ in their developmental rates. Our results indicate that the progression of patterning is coordinated with some, but not all, developmental events and varies between events.

## Results

### Developmental Staging Scheme for Wing Discs

**Figure 2:**
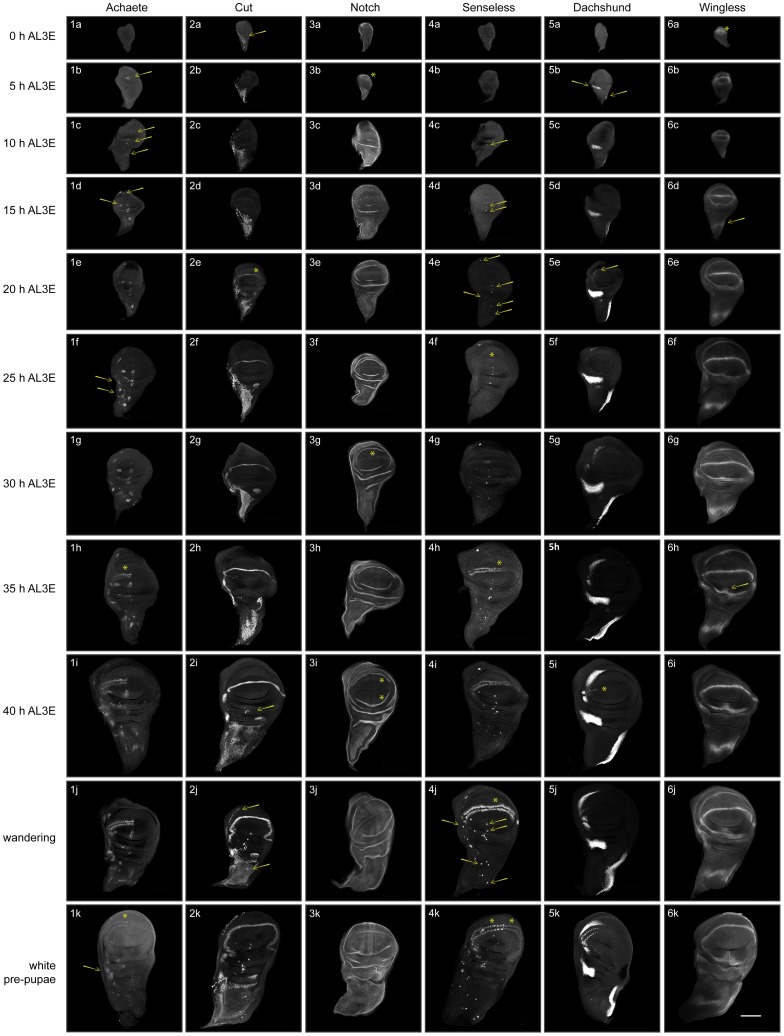
Patterning progression of six of the eleven gene products used to construct the staging scheme. The expression of Achaete (1a-1k), Cut (2a-2k), Notch (3a-3k), Senseless (4a-4k), Dachshund (5a-5k) and Wingless (6a-6k) at 0 (1a-6a), 5 (1b-6b), 10 (1c-6c), 15 (1d-6d), 20 (1e-6e), 25 (1f-6f), 30 (1g-6g), 35 (1h-6h) and 40 (1i-6i) hours after third instar ecdysis (h AL3E), wandering (at the average time of 46 h AL3E, 1j-6j) and white pre-pupae (at the average time of 49 h AL3E, 1k-6k). Arrows show addition or change of cells or patches of cells, and asterisks highlight changes in stripes. Scale bar is 100 µm.

To compose our developmental staging scheme for wing discs, we used immunocytochemistry to identify changes in the expression of eleven patterning gene products at five hour intervals from 0–40 h after third instar ecdysis (AL3E), at wandering, and at pupariation for a total of eleven time points (Figure 2 and Supplementary Figs S1, S2, S3). We used wing discs from larvae of an isogenic wild-type strain Samarkand (SAM) reared at 25°C (wild type at 25°C). Three of these time points coincided with three developmental events – the moult to the third instar, wandering and pupariation. We have a strong understanding of the physiology underlying these developmental events, and so assaying patterning at these time points allowed us to test for coordination between tissue patterning and whole-body development. Collectively, we used the progression of patterning in wild type at 25°C as a baseline for all comparisons in this work.

**Figure 3:**
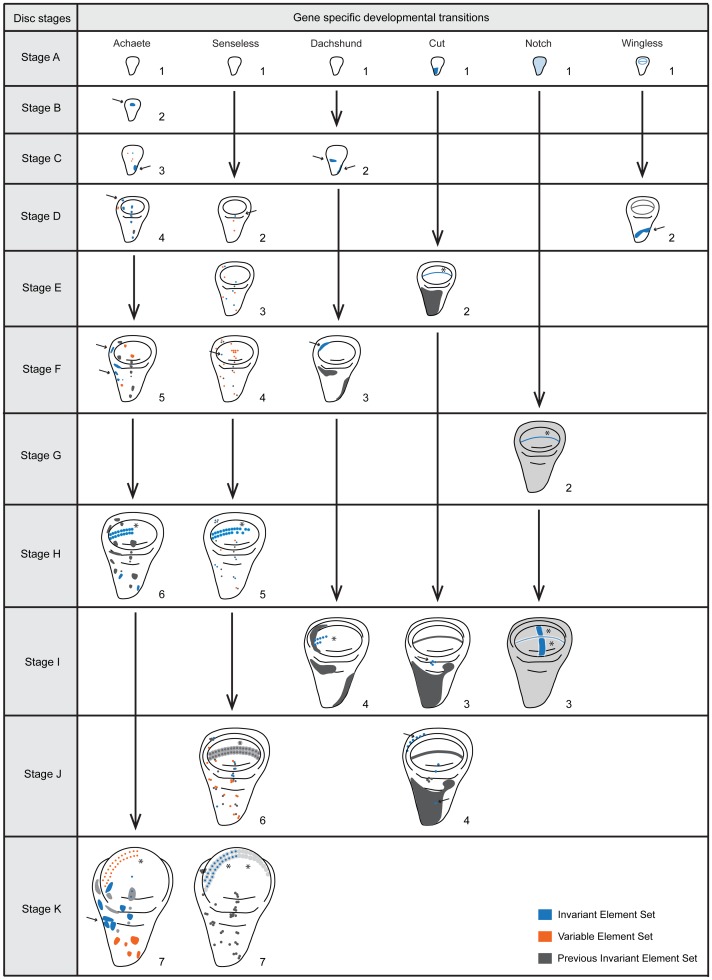
Staging scheme – the developmental transitions for each gene product arranged according to disc stage. Each column represents a gene product (Achaete, Senseless, Dachshund, Cut, Notch and Wingless) and each row a disc stage (A-K). We characterized disc stages by the combination of gene-specific stages (numbers under each disc for each gene). We highlighted the key elements characterizing each gene-specific stage either with arrows (cells or patches of cells) or asterisks (stripes). For Dachshund (Dac) stage 4 where the stripe is highlighted, we did not consider the length of the stripe for this character, although it increases in length during development. Further, in each disc, in blue we represent addition or change of elements that are common to all discs sampled (Invariant Element Set), in orange we represent the addition or change of elements that are variable (Variable Element Set, ie. do not appear in all the discs sampled) and in grey the elements that do not change in comparison to the previous gene stage (Previous Invariant Element Set). We only used the invariant element set to construct the staging scheme. Black vertical arrows represent the transition between the gene-specific stages. Disc stages A-K correspond to the time points sampled (0, 5, 10, 15, 20, 25, 30, 35, 40, 46 (average time for wandering) and 49 (average time for pupariation) hours after third instar ecdysis (h AL3E).

We identified elements of pattern that we could reliably distinguish across discs of a given time point (Figs 2, 3). New elements of pattern included the addition of a new region of expression, for instance the appearance of expression in a cell or in cells that previously had not expressed a particular gene product; the refinement of an expression field from diffuse expression in a group of cells to more focussed expression in a reduced subset of cells; or the disappearance of expression in a region that had previously expressed that gene product. For each patterning gene product, we discerned the time each patterning element arose, thereby characterizing the transitions in pattern for each gene. From this, we defined stages for each gene product (referred to as gene-specific stages) (Figure 3).

Not all gene products displayed clear gene-specific stages. Engrailed and Patched did not undergo patterning transitions in the third larval instar, consistent with previous studies [17]. Scabrous localization within single cells appeared to be restricted to vesicles, making changes in pattern hard to identify. Hindsight expression in the wing disc was difficult to distinguish from expression in associated tracheal cells. Finally, the patterning transitions for Delta and Notch (N) occurred at the same time. For these reasons, we chose to exclude Engrailed, Patched, Scabrous, Hindsight, and Delta from our characterizations of overall disc stage.

We tabulated the gene-specific stages for each time point from the remaining six gene products, Achaete (Ac), Ct, N, Sens, Dachshund (Dac) and Wg. These combinations of gene-specific stages allowed us to define eleven disc stages (A-K), corresponding to each of the eleven time points sampled from wild-type larvae at 25°C (Figure 3 and see Materials and Methods).

**Figure 4:**
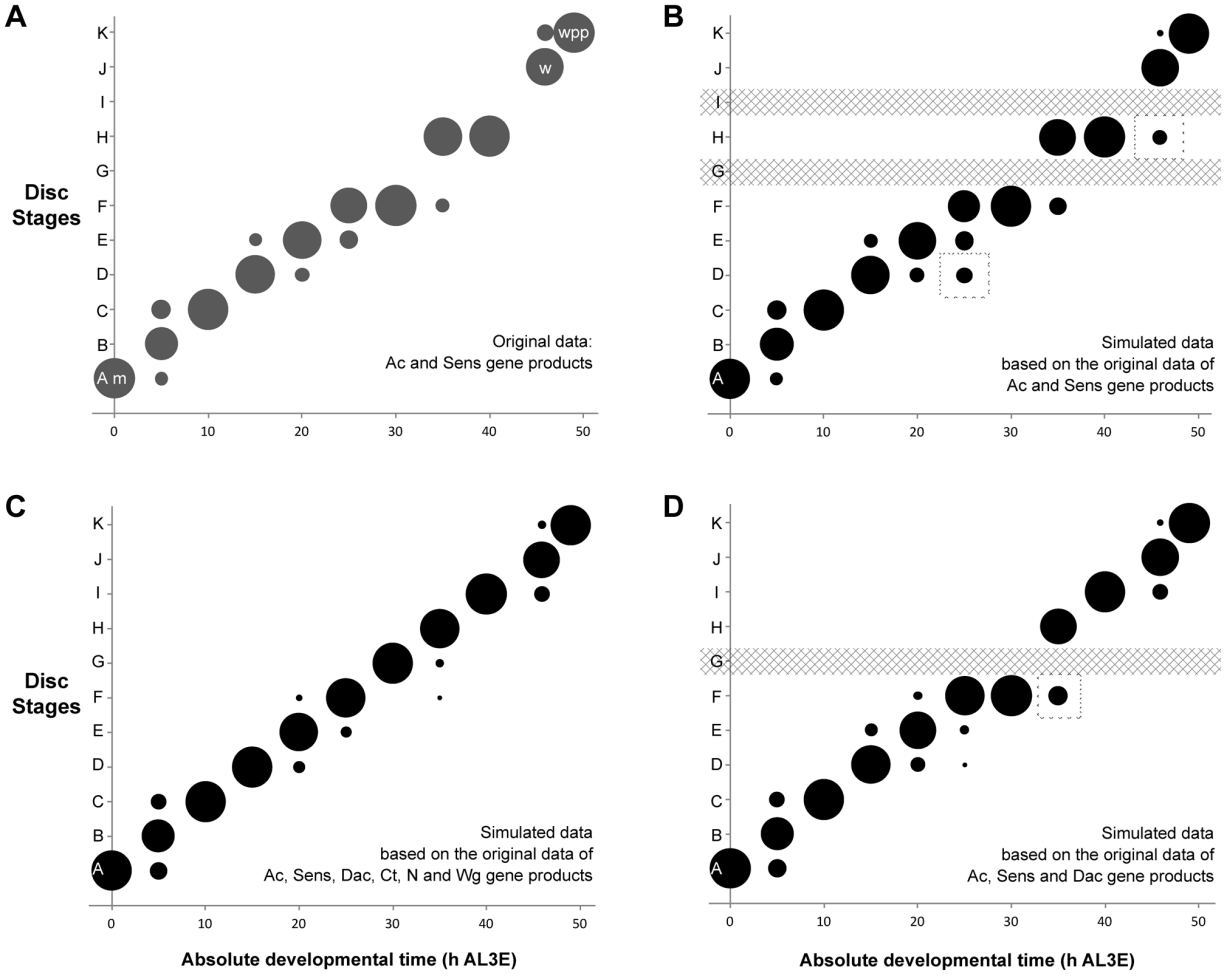
The probability of attributing a wing disc dissected at a given age to a particular disc stage. (a) Proportion of discs attributed to each disc stage based on individual discs simultaneously staged for Ac and Sens. (b) By applying the Naïve Bayes Classifier (NBC) to the permuted data set based only on Ac and Sens expression patterns, our staging scheme was able to distinguish between discs from each time point and classify them into their appropriate stages. Dashed boxes highlight regions where the simulated data set showed greater variation than the actual data from a. (c) We repeated the NBC classifier analysis using the expression patterns of Ac, Sens, Dac, Ct, N and Wg. (d) Using the expression patterns of Ac, Sens and Dac to classify the discs, the results were nearly identical, except the NBC classified all discs at 30 h AL3E (stage G) as stage F. This is because stages G and F share the same Ac, Sens and Dac expression patterns. The dashed box marks a time point where the simulated data from Ac, Sens and Dac alone showed greater variation than the simulated data generated using the complete panel of six patterning gene products. Developmental events are identified by m (moult to the third instar), w (wandering) and wpp (white pre-pupae – pupariation).

Two of the gene products, Ac and Sens were staged simultaneously in individual discs (Ac is a mouse monoclonal antibody and Sens is a guinea pig antibody). Using these two gene products alone, we can assign discs to nine of the eleven disc stages (Figure 4a). The bubbles in Figure 4a represent the proportion of discs at each time point that fall into a particular disc stage based on their Ac and Sens pattern combined. These data show that using Ac and Sens alone, for five time points all discs are categorized into a single stage. For the remaining six time points sampled, most discs (67–89%) can be attributed to one disc stage, with a smaller proportion of discs (<24%) falling into one or two additional stages. Thus, staging with Ac and Sens alone provides a reliable measure of disc stage across developmental time.

We expected that adding more markers to our staging scheme would increase its resolution. Problematically, due to the nature of antibody staining, it was not possible to stain a single disc for more than two gene products. Consequently, we cannot assign an individual disc to a particular developmental stage with the complete set of markers. To circumvent this problem, we simulated what a disc would look like if we could stain the same disc for all six gene products. We first tabulated the observed stages for each gene product at each time point. The number of discs scored for each gene product ranged from five to sixteen (Supplementary Table S1), depending on the time point and the gene product. We then randomly sampled from this table to simulate all the possible combinations of gene-specific stages for a single disc dissected at this time point. We repeated these permutations 1000 times to generate 1000 simulated discs for each time point. We then applied a Naïve Bayes Classifier (NBC) to the simulated data set to assign each simulated disc to a developmental stage, based on our staging scheme. The NBC analysis does not return a p-value, but instead provides the probability that a disc of a given time point would be assigned to a particular disc stage.

The results of this analysis are represented using a bubble plot (Figure 4b-d). In this plot, the area of each bubble is the proportion of the 1000 simulated discs that were assigned to each disc stage, using the NBC. As a proof of principle, we applied our analysis to the staging scheme devised from the Ac and Sens data. The plot generated from the simulated discs looks very similar to the staging scheme derived from the sampled disc data (Figure 4a, b), although the NBC appears to slightly overestimate the amount of variation in the data (dashed boxes in Figure 4b). Overall, however, our stimulated data set represents well the patterns seen from the sampled discs.

Next, we simulated discs with all six patterning gene products and applied the NBC (Figure 4c). Using all six gene products, we could resolve eleven disc stages in the simulated discs. For six time points, there is a single bubble, indicating that all the simulated discs at that time point share a stage-specific combination of gene-product patterns. This suggests that the criteria for classification are unambiguous at that time point. In the remaining time points, the NBC assigned discs to two or three stages. This indicates that the discs dissected at these time points did not all share the characteristics used to define a single stage. That is, there is variability in patterning among discs dissected at the same time point. Nevertheless, even at these time points the NBC classified the majority of simulated discs (65–94%) to a single stage. Further, the amount of variation for these time points was reduced if the complete data set was used in the simulation instead of using Ac and Sens alone.

We repeated the NBC analysis using only the expression patterns of Ac, Sens and Dac to classify the discs. The results were nearly identical from the complete gene set simulations (Figure 4d), except the NBC classified all discs at 30 h AL3E (stage G) as stage F. This is because stages G and F share the same Ac, Sens and Dac expression pattern, and so the NBC classified the discs into the earliest stage by default. This combination of three gene products provides greater resolution than Ac and Sens alone and was one of the combinations that identified most of the disc stages from the moult to the third instar until pupariation. Hereafter, to minimize the number of gene products necessary to stage wing discs, we established the staging scheme composed from Ac, Sens and Dac as the baseline for all subsequent comparisons. Additionally, we choose to use Wg for the first time point because Ac, Sens and Dac were not expressed at the moult to the third instar.

### Changing the Rate of Development by Modifying Environmental Conditions: The Effects of Temperature

**Figure 5:**
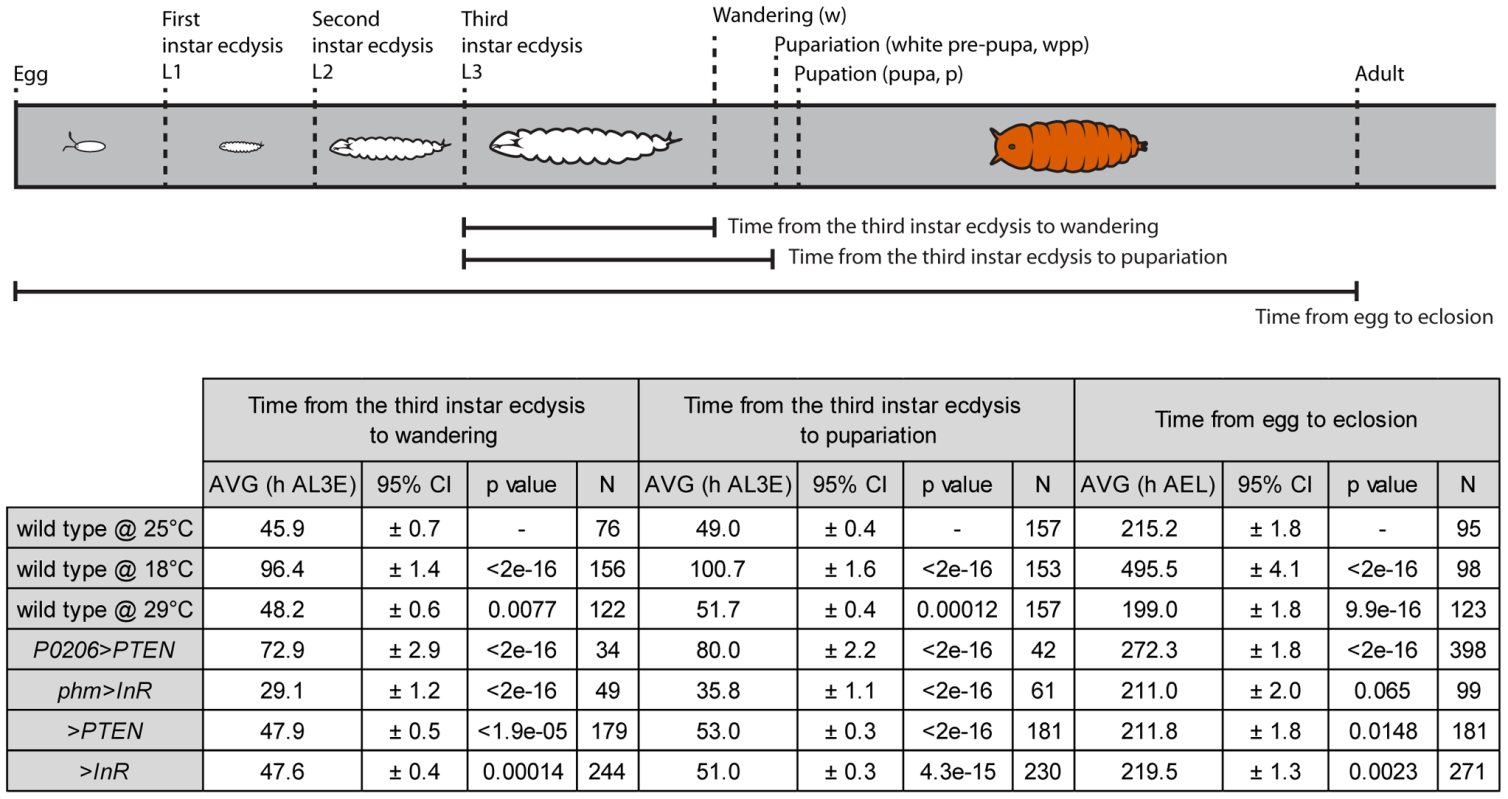
Schematic representation and table of time from the third instar ecdysis (L3) until wandering, pupariation and time from egg to eclosion. Developmental times are represented in hours after third instar ecdysis (h AL3E), hours after egg lay (h AEL) and characterized by mean (AVG) and 95% confidence intervals (CI). Data was tested for normality using Q-Q plots and analyzed using one-way ANOVA (α = 0.025). P values are from pairwise t-tests, and refer to differences between the temperature treatments (wild type at 18°C and 29°C compared to 25°C), differences between physiological treatments and wild type at 25°C (*P0206>PTEN* and *phm>InR* versus wild type at 25°C) and differences between parental backgrounds (*>PTEN* and *>InR*) and wild type at 25°C.

Once we had a method of defining the developmental stage of a disc, we then asked whether the progression of pattern through these developmental stages was tightly coordinated with whole-body development when developmental rate was altered by changes in rearing temperature. Rearing wild-type larvae at 18°C lengthened the time to adult eclosion from larval hatching, while rearing larvae at 29°C shortened the time, compared to wild-type larvae raised at 25°C (Figure 5). Surprisingly, however, the duration of the third larval instar was slightly longer at 29°C than at 25°C (Figure 5), as was the time to larval wandering from the beginning of the third instar. Thus, for the purposes of our study, larvae reared at 29°C were slow developers.

**Figure 6:**
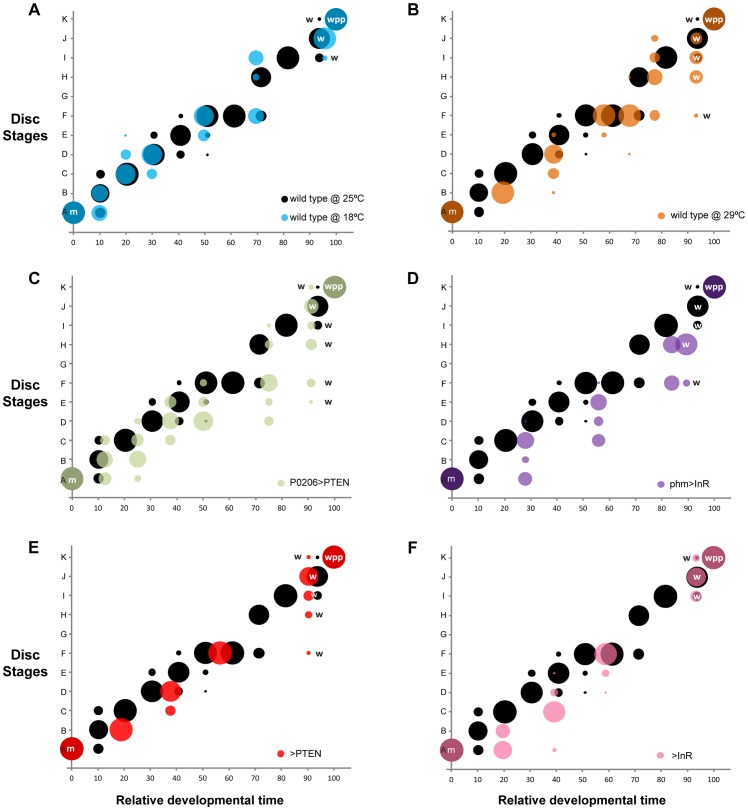
Changing developmental time alters the progression of pattern. Probability (represented by the size of the circle) that a disc with a given set of gene-specific stages belongs to a particular disc stage, varying with relative developmental time (normalized to pupariation). In all panels (a-f), we show the wild type at 25°C in black. (a-b) Temperature manipulations: (a) disc stages attributed to discs from wild-type larvae reared at 18°C are shown in blue and (b) from wild-type larvae reared at 29°C are shown in orange. (c-d) Manipulations of the timing of ecdysis synthesis: (c) disc stages attributed to discs from *P0206>PTEN* larvae are shown in green and (d) disc stages attributed to discs from *phm>InR* larvae are in purple. (e-f) Parental lines to test for the contribution of genetic background: (e) disc stages attributed to discs from *>PTEN* larvae in red and (f) from *>InR* larvae in pink. Developmental events are identified by m (moult to the third instar), w (wandering) and wpp (white pre-pupae – pupariation).

To assay whether the progression of disc patterning relative to whole-body development was affected by rearing temperature, we used a bubble plot to chart wing disc stage, as assigned by the NBC classifier applied to a permuted data set, expressed in relative developmental time (normalized to pupariation), at 18°C, 25°C and 29°C. At all three temperatures, patterning in the discs was the same at the moult to the third instar and at pupariation (Figure 6a, b and Supplementary Figure S4a, b). At 18°C the progression of disc patterning when normalized to pupariation time was largely the same as at 25°C, indicated by the overlapping bubble plots at the two temperatures (Figure 6a and Supplementary Figure S5). In contrast, at 29°C patterning was initially delayed, evident from discs dissected at the same relative developmental time showing earlier patterning stages at 29°C than at 25°C (Figure 6b and Supplementary Figure S4b). The rate of patterning progression accelerated later in the third instar, however, to achieve the final disc stage at pupariation (Figure 6b and Supplementary Figure S4b). Further, there was more variation in developmental stage among discs dissected at larval wandering at 29°C, compared to 25°C (Figure 6b). Earlier in development, the variation and delay observed in disc stage at 29°C was due to Ac and Sens expression, both of which belong to the Notch signalling pathway (Supplementary Figure S6). In contrast, at wandering much of the delay was caused by variation observed in Sens and Dac expression patterns (Supplementary Figs S6, S7).

### Changing the Rate of Development by Modifying Larval Physiology: Altering the Timing of Ecdysone Synthesis

The timing of ecdysone synthesis is thought to be key to coordinating whole-body developmental events (moulting, larval wandering and pupariation) with imaginal disc development. To test this hypothesis, we first altered the timing of ecdysone synthesis by downregulating or upregulating insulin signalling in the prothoracic gland, lengthening or shortening the duration of the third larval instar respectively (Figure 5) [17]. To downregulate insulin signalling in the prothoracic gland, we used the *P0206 GAL4* driver to overexpress PTEN (*P0206>PTEN*); to upregulate insulin signalling in this tissue, we expressed InR using the *phm GAL4* driver (*phm>InR*). Together with changes in the duration of development, the rate of patterning in the wing discs was also affected. Early in the third larval instar, patterning appeared to be retarded in both *phm>InR* and *P0206>PTEN* larvae, while patterning progressed at an accelerated rate later in development (Figure 6c, d and Supplementary Figure S4c, d).

To explore how wing disc patterning progressed relative to whole-body development, we again used a bubble plot to chart wing disc stage in *phm>InR* and *P0206>PTEN*, as assigned by the NBC classifier applied to a permuted data set, against relative developmental time. We used wild-type SAM larvae reared at 25°C for comparison. Under all experimental conditions, wing discs displayed the same pattern at the beginning (moulting) and end (pupariation) of the third larval instar. However, a bubble plot of relative developmental time (normalized to pupariation) against disc stage indicated that in both *P0206>PTEN* and *phm>InR* larvae, disc patterning is initially delayed and showed increased variability compared to 25°C wild-type larvae at the same relative developmental time (Figure 6c, d and Supplementary Figs. S4, S8, S9). This delay is more evident in *phm>InR* discs (Figure 6d), where it is due to changes in the relative progression of Ac, Sens and Dac expression (Supplementary Figure S9), than in *P0206>PTEN* discs (Figure 6c), where it is primarily due to changes in the progression of Ac and Sens expression (Supplementary Figure S8). Furthermore, in *phm>InR* discs from wandering larvae, patterning was substantially delayed when compared to wild type at 25°C (Figure 6d).

Some of the observed changes in wing disc patterning progression early in the third instar in *P0206>PTEN* and *phm>InR* larvae may result from genetic background effects. Both parental lines, *yw; UAS PTEN* (referred to as *>PTEN*) and *yw flp; UAS InR29.4* (referred to as *>InR*), showed small but significant differences in pupariation time compared to the wild type at 25°C (Figure 5). Additionally, in both *>PTEN* and *>InR* larvae, we observed early delays in wing patterning relative to wild type at 25°C, due to retardation in the progression of all three gene products – Ac, Sens and Dac (Supplementary Figs S4e, f, S10, S11). However, after 50% developmental time wing disc patterning was the same in all three lines (wild type at 25°C, *>PTEN*, *>InR*). Further, wing disc patterning was the same in all three lines at moulting and pupariation, and largely overlapped at wandering (Figure 6e, f). A comparison of wing disc patterning in *P0206>PTEN* and *phm>InR* larvae to their genetic controls suggests that the delays observed before 50% relative developmental time are due to genetic background effects while the delays after this period are due to changes in physiology (Supplementary Figure S12).

### Examining the Correlation between Gene-Specific Stages from Two Genes in the Same Patterning Cascade

**Figure 7:**
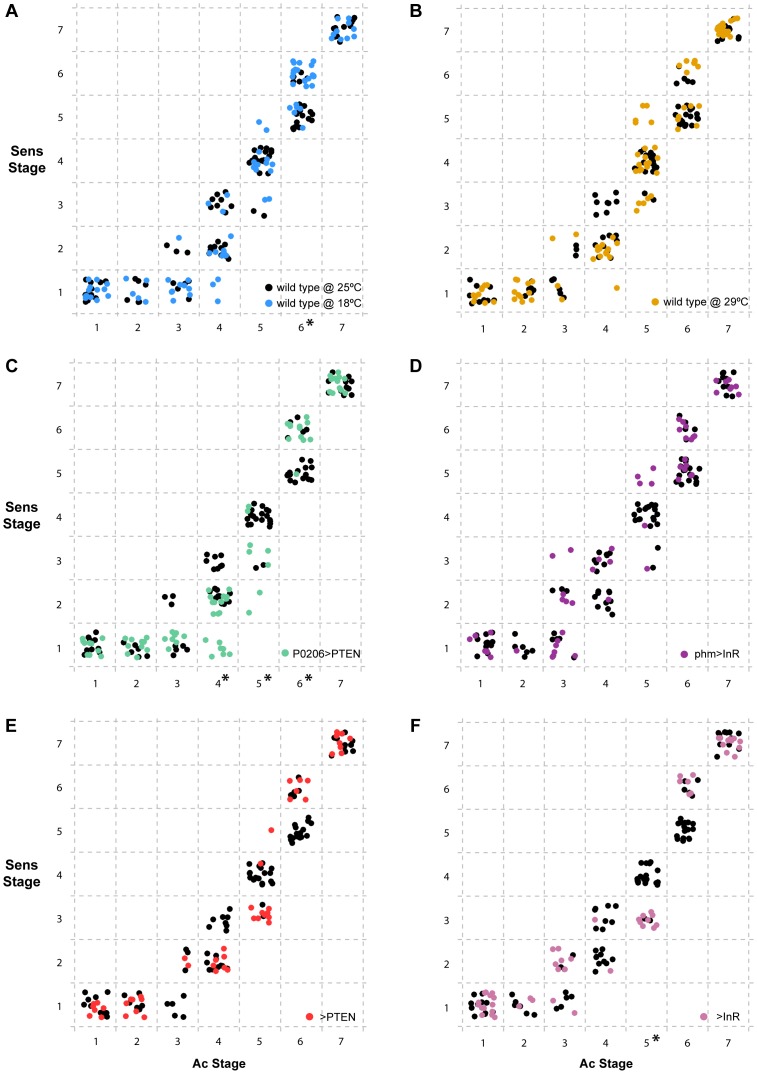
Progression of Senseless (Sens) stage as a function of Achaete (Ac) stage independent of developmental time. For each individual disc sampled, we represented the combination of Ac and Sens stages observed. Inside dashed boxes, all discs were assigned the same discrete Sens and Ac stage. In all panels (a-f), we show the wild type SAM larvae at 25°C in black. (a) discs from wild type SAM larvae reared at 18°C are shown in blue and (b) from wild type SAM larvae reared at 29°C are shown in orange. (c) discs from *P0206>PTEN* larvae are shown in green and (d) from *phm>InR* larvae are in purple. (e) disc-specific stages attributed to discs from *>PTEN* larvae in red and (f) from *>InR* larvae in pink. Ac stages with asterisks (on the x-axis) are those that show significant differences between conditions/genotypes (p<0.01, Wilcoxon rank test using Holm's p-value adjustment) with reference to the wild type SAM at 25°C.

Our data demonstrate that altering developmental timing of the whole body changes the progression of patterning in Ac, Sens and Dac. Next, we explored whether gene-specific stages of Sens correlated with gene-specific stages of Ac across treatments and genotypes independently of developmental time (Figure 7). We found that overall, Ac and Sens stages were tightly correlated and showed little significant variation with temperature, physiology or genotype. There were some exceptions; for Ac stages 4 and 5 we found that Sens stages were significantly delayed in *P0206>PTEN* larvae when compared to wild-type larvae at 25°C (Figure 7c). The *>InR* larvae showed similar delays in Sens with respect to Ac at stage 5 (Figure 7f). In contrast at Ac stage 6, Sens was accelerated in the wild-type larvae at 18°C and in the *P0206>PTEN* larvae (Figure 7a, c). Thus, Sens stages show some degree of plasticity with respect to Ac stages, but only at Ac stages 4–6.

## Discussion

In this study, we set out to examine the extent to which tissue development is coordinated with the development of the whole body. We tested two alternative hypotheses: 1) the progression of pattern is tightly coordinated with whole-body development at all times, and 2) patterning is coordinated only at developmental milestones.

Previous studies demonstrated that the development of tissues could regulate the timing of whole animal development. Specifically, larvae with slow growing discs greatly delay the development of the whole body [26]–[29]. Discs induce these delays by regulating the timing of a specific developmental event that occurs early in the third instar, termed critical weight [26], [28]. Slowing disc growth after critical weight has no effect on developmental timing [28]. Delaying patterning in the imaginal discs has also been shown to retard the development of the whole body. If the spread of Wg protein is restricted in the imaginal discs by replacing wild-type Wg with a membrane-tethered Wg allele, larvae delay the onset of pupariation [30]. We do not yet know whether Wg signalling in the discs affects developmental timing by affecting disc growth rate nor do we know which developmental events are affected by altered Wg signalling.

Further, there is ample evidence from many insects that ecdysone controls the timing of development in the various tissues of the body [13]. In third instar larvae, ecdysone signalling stimulates neurogenesis in the optic lobe via the Notch/Delta pathway [31]. The pulses of ecdysone that stimulate the onset of pupal development are also known to initiate patterning of the sensory tissues of the wing [16]. Thus, it seemed likely that ecdysone pulses at other stages could act as milestones to coordinate both tissue and whole-body development.

We found that patterning, as determined by disc stage, aligned at the moult to third instar and at pupariation in all conditions studied. It is important to note, however, that considerable patterning occurs in wing discs before the third instar [32]. Furthermore, pupariation is not an endpoint for disc pattern, as the patterning of sensory structures and the specification of the wing veins continue on during pre-pupal and pupal development [15], [16], [33]. Thus pupariation appears to be characterized by an alignment but not termination of patterning progression.

In contrast, disc patterning among wandering larvae showed variability, both within the wild type at 25°C and across experimental treatments. Variation in disc stage at wandering within the reference genotype at 25°C is likely to be due to the fact that the wandering stage lasts approximately 8 hours and therefore occupies a slightly longer time interval than the other intervals of the staging scheme. This, however, does not explain the difference in disc stage at wandering across experimental treatments; discs from *phm>InR* larvae were mostly at disc stage H at wandering, whereas the wild-type discs at 25°C were mostly at disc stage J. Thus, we conclude that wing patterning is not coordinated with whole-body development at wandering. This was surprising, as wandering is commonly used to stage larvae to ostensibly the same developmental point (for examples see [34]–[36]). Overall, our data supports hypothesis two: patterning aligns with whole-body development at specific developmental milestones, the moult and pupariation, and shows greater variation between these milestones.

Variability in pattern between the moult and pupariation showed common characteristics across treatments and genotypes. Generally, patterning showed delays relative to whole-body development early in the third instar. Disc patterning accelerated relative to whole-body development towards the end of the third instar to reach the final stage at pupariation (Figure 6 and Supplementary Figure S4). Our data highlight the possibility that because perturbations in pattern occur through delays early in the third instar, there is an intrinsic checkpoint late in the third instar that regulates pattern in the discs so that they reach a common patterning stage at pupariation.

The progression of pattern also varied with genetic background. This variation between control genotypes was most apparent early in development. In contrast to the environmental/physiological treatments, patterning was, however, aligned at wandering. This observation suggests that our staging scheme would vary somewhat with the genotype chosen as the reference background. Genetic variation in the mechanisms controlling developmental robustness has been previously described in the context of evolutionary studies. For instance, in *Caenorhabditis elegans* the types of deviations observed during the highly robust process of vulval development depend on genetic background [37]. We expect that genetic variation in the progression of patterning systems is common, but that it is often undetected due to alignment at developmental milestones.

Many of the delays in the progression of pattern that we observed across developmental time were due to delays in two genes from the same pathway, Ac and Sens [38], [39]. This likely reflects the observation that the progression of patterning in these two genes was correlated, independent of developmental time. Consequently, when one gene was delayed, so was the other. In contrast, delays in Dac expression tended to occur at later stages of development. Taken together, this raises the question of how environmental perturbations might affect gene expression within or between signalling pathways as an interesting avenue for future study.

Collectively, our data reveal that tissue patterning is coordinated with some but not all whole-body developmental events. This raises two questions: first, across all of development which whole-body developmental events are developmental milestones for tissues? Second, do all tissues align their development to the same milestones?

Because many developmental events are regulated by ecdysone, whether or not a tissue aligns its pattern to a particular developmental event may be due to its sensitivity to ecdysone at that time. The response of a tissue to a given ecdysone pulse is likely to be tied to its function. If we had examined the development of tissues that have functions in the larvae, we might have found tighter coordination with wandering. For example, the pulse of ecdysone that initiates larval wandering also coordinates the onset of autophagy in the fat body [40]. Autophagy in this tissue is thought to sustain the growth and development of other tissues during non-feeding stages [41]. In the salivary glands, a pulse of ecdysone in the mid-third instar stimulates glue production, while the pulse at larval wandering induces movement of the glue from the cells into the lumen of the gland [42]. This glue is then expelled in response to the ecdysone pulse at pupariation to cement the animal to the substrate. Consequently, development of the fat body and salivary glands may be tightly coordinated with larval wandering. In contrast, tissues like the imaginal discs, whose differentiation into their adult form only starts after pupariation, may not need to respond to these earlier ecdysone pulses.

Despite the striking effects that environmental and physiological changes induce in developmental timing, the resulting adults bear correctly patterned structures. We originally presumed that this was because developmental time and patterning of the tissues was tightly coordinated. Using our staging scheme, however, we have shown that patterning and whole-body development are coordinated only at moulting and pupariation, suggesting these events mark milestones during development. A third event, wandering, does not appear to act as a developmental milestone, at least as far as wing disc patterning is concerned. We also found that the progression of pattern in the wing disc is far more plastic than originally supposed. Further, we found that both the duration of developmental intervals and rates of patterning can be slowed down or sped up. Thus underlying the robustness of the adult phenotype, we have revealed that developmental milestones coordinate wing disc and whole-body development to cope with environmental and physiological variation.

## Materials and Methods

### Fly Stocks and Rearing Conditions

We used an isogenic wild-type strain, Samarkand (SAM), reared at 25°C to develop the staging scheme, representing the baseline for all comparisons (referred to as wild type at 25°C). To manipulate developmental time environmentally, we reared wild-type SAM flies at 18°C and 29°C (wild type at 18°C and wild type at 29°C). To alter the timing of ecdysone synthesis and manipulate developmental time physiologically, we used the progeny from *phm*-GAL4 crossed with yw *flp; UAS InR29.4* (*phm>InR*) and from *P0206-GAL4* crossed with *yw; UAS PTEN* (*P0206>PTEN*) to up- or down-regulate insulin signalling in the prothoracic gland, respectively. Even though *P0206*-GAL4 is a weaker GAL4 driver for the prothoracic gland and also drives expression in the corpora allata, we chose to use it to drive *UAS PTEN* because *phm>PTEN* larvae die as first instar larvae [25]. We used the parental lines *yw; UAS PTEN* (*>PTEN*) or *yw flp; UAS InR29.4* (*>InR*) as additional controls for genetic background effects.

Flies were raised from timed egg collections (2–6 hours) on standard cornmeal/molasses medium at low density (200 eggs per 60×15 mm Petri dish) in a 12 h light-dark cycle with 70% humidity, and maintained at 25°C unless stated otherwise. Larvae that were reared at 18°C or 29°C were maintained in incubators without lights due to equipment constraints.

### Animal Staging and Developmental Time

Larvae were staged into 1-hour cohorts at ecdysis to the third larval instar and wing-imaginal discs were dissected at the following times (in h AL3E): wild type at 25°C: 0, 5, 10, 15, 20, 25, 30, 35, 40, 46 (wandering) and 49 (pupariation) h AL3E; wild type at 18°C: 0, 10, 20, 30, 50, 70, 96 (wandering) and 101 (pupariation) h AL3E; wild type at 29°C: 0, 10, 20, 30, 35, 40 h, 48 (wandering) and 52 (pupariation) h AL3E; *P0206>PTEN*: 0, 10, 20, 30, 40, 60, 73 (wandering) and 80 (pupariation) h AL3E; *phm>InR*: at 0, 10, 20 and 30 h AL3E, 32 (wandering) and 36 (pupariation) h AL3E; *>PTEN* control: 0, 10, 20, 30, 48 (wandering) and 53 (pupariation) h AL3E; >*InR* control: 0, 10, 20, 30, 48 (wandering) and 51 (pupariation) h AL3E.

We measured the average time to wandering and pupariation by counting the number of larvae wandering/pupariating within a cohort every two hours. To measure the average eclosion time, we allowed flies to oviposit for 2–6 hours in food bottles. Larvae were maintained at low densities, and we checked for adult eclosion every 12 h.

### Dissections and Immunocytochemistry

To develop our staging scheme, we examined the expression of eleven patterning gene products in the wing discs of wild-type larvae at 25°C by immunocytochemistry: Achaete (Ac), Cut (Ct), Delta (Dl), Hindsight (Hnt), Notch (N), Scabrous (Sca), Senseless (Sens), Dachshund (Dac), Engrailed (En), Patched (Ptc) and Wingless (Wg). These patterning gene products represent the main cascades involved in wing disc patterning: the Notch signalling pathway (represented by Ac, Ct, Dl, Hnt, N, Sca and Sens), the Hedgehog signalling pathway (represented by Dac, En and Ptc) and the Wnt/Wg signalling pathway (represented by Wg). In the wing discs of larvae with altered developmental time (wild type at 18°C, wild type at 29°C, *P0206>PTEN* and *phm>InR*) as well as the genetic controls (*>PTEN* and *>InR*), we examined the expression of four gene products: Wg for the 0 h AL3E time point, and Ac, Sens and Dac for all time points. Although it was impossible to simultaneously stain for all gene products at all time points for all genotypes under all conditions, we minimized the effects of variation between experimental blocks by conducting experiments between at least two genotypes/conditions in parallel. Further, for any given time point for each of the genotypes/conditions, we stained for different patterning gene products on different days.

For each time point, wing imaginal discs from 10 larvae were dissected in cold phosphate buffered saline (PBS) and fixed for 30 min in 4% paraformaldehyde in PBS. Number of dissected discs varies from 5–16 depending on the treatment/genotype (Supplementary Tables S1 and S2). The tissue was washed in PBT (PBS +1% Triton X-100) at room temperature, blocked in PBT-NDS (2% Normal Donkey Serum in PBT) for 30 min and then incubated in a primary antibody solution (Supplementary Table S3) overnight at 4°C. After washing with PBT, tissue was incubated with fluorescently-conjugated secondary antibody overnight at 4°C. Tissue was rinsed with PBT and wing discs were mounted on a poly-L-lysine-coated coverslip using Fluoromount-G (SouthernBiotech). Samples were imaged using a Zeiss LSM 510 confocal microscope and images were processed using ImageJ.

### Qualifications and Quantifications Used to Characterize Gene Product Patterns

The expression patterns of each of the gene products examined had previously been characterised in the literature: individual cells, patches of cells, or stripes [34], [43]–[45] (Supplementary Figure S1). To compose the staging scheme, we initially conducted a qualitative analysis of the patterns observed for each gene product at each time point (Figure 2 and Supplementary Figure S2) and described their progression. We then quantified these expression patterns in two ways (Supplementary Figs S2, S3). First, we divided the area of gene product expression by the total area of the disc, to generate a measure of pattern area. Second, we quantified the number of specific elements (cells, patches of cells or stripes) that each expression pattern exhibited. By both quantifying gene product expression and characterising the addition of new pattern elements through time, we were able to identify the gene products that varied the most during the third instar as well as those patterning elements that changed through a stepwise progression. We then used the change in patterns of these gene products to generate a staging scheme.

### Statistical Analysis

We used a Naïve Bayes Classifier (NBC) to test the power of our staging scheme to classify dissected discs from each time point into their correct stage. We first tabulated the observed gene-specific stages for all the patterning-gene products in the dissected discs from each time point. We then permuted the data from each time point 1000 times to simulate a population of 1000 discs with the range and frequency of gene-specific stages that was characteristic of wing discs from that time point. We then trained an NBC using our staging scheme and applied it to the permuted data set to determine what proportion of the 1000 simulated discs from each time point would be classified into the ‘correct’ stage. We repeated this analysis to assign stages to discs dissected from larvae reared under all experimental conditions.

All data analyses and statistics were conducted using R. The R scripts used to analyse the data, as well as the complete data, are available for download from Dryad (doi:10.5061/dryad.fq134).

## Supporting Information

Figure S1 Definitions of patterning elements used to characterize stages in the staging scheme. (a) The third instar wing disc is already subdivided into domains that will form the wing pouch, wing hinge and notum of the adult fly. It has an anterior (A) and posterior (P) axis and dorsal (D) and ventral (V) domains. (b) The element *cell* was defined by one round dot of expression, which corresponded to the refinement of expression to a sensory organ precursor (SOP). For later time points, SOPs divide giving rise to two sister SOPs, referred as doublets (arrow). (c) *Patch of cells* refers to a region of pattern that resembles a cluster of cells, either clearly delimited or diffuse. (d-g) *Stripe* corresponds to one or more line of cells (more or less defined) (d, e), that can be parallel (double stripe) or perpendicular (forming a cross) to each other, located in the developing wing pouch along the dorsal-ventral axis. Stripes also appear as lines restricted to the anterior side of the wing pouch or along the dorsal-ventral boundary with a surrounding ring (stripe with ring, f), or along the anterior-posterior axis (g). The stripes correspond to lines of SOPs along the wing margin (e), or lines of positional information regarding wing disc boundaries (d). Different compositions of these elements describe all observed patterns for each gene product through development.
(TIF)

Figure S2 Patterning progression of three of the eleven gene products initially assessed but not included in the staging scheme. Dynamic expression of Delta (1a-1j), Hindsight (2a-2j) and Scabrous (3a-3j) at 0 (1a-3a), 5 (1b-3b), 10 (1c-3c), 15 (1d-3d), 20 (1e-3e), 25 (1f-3f), 30 (1g-3g), 35 (1h-3h), and 40 (1i-3i) hours after third instar ecdysis (h AL3E) and wandering (1j-3j). Arrows show addition or change of cells or patches of cells, and asterisks highlight changes in stripes. (1f) Arrows highlight Delta expression mainly in the hinge and notum. Hindsight undergoes four transitions adding new elements at 15, 25 and 35 h AL3E. Lastly, Scabrous undergoes four transitions adding new elements at 10, 15 and 40 h AL3E. (3h) shows Scabrous expression in the centre of the wing pouch, before it refines to a stripe. (Scale bar 100 µm). (k-o) Quantitative measures of the relative amount of expression normalized to disc size of the different elements observed. (k) Delta expression pattern represented by the progression of the stripe. (l-m) Hindsight expression pattern decomposed into (l) pattern area and (m) progression of the stripe. (n-o) Scabrous expression pattern decomposed into (n) pattern area and (o) progression of the stripe. Delta undergoes four transitions in its pattern, adding new elements at 5, 35 and 40 h AL3E.
(TIF)

Figure S3 Quantitative measures of the relative amount of expression normalized to disc size and of the different elements observed for six of the eleven gene products. (a-c) Achaete expression pattern decomposed into (a) pattern area, (b) number of patches of cells and (c) progression of the stripe. (d-f) Cut pattern broken down into (d) pattern area (whole disc, only notum and hinge, and only wing pouch), (e) number of patches of cells and (f) progression of the stripe. (g) Notch expression pattern represented by the stripe progression. (h-k) Senseless expression pattern decomposed into (h) pattern area, (i) number of SOPs, (j) progression of the stripe and (k) number of doublets. (l-n) Dachshund expression pattern in terms of (l) pattern area, (m) number of patches of cells and (n) progression of the stripe. (o) Wingless pattern area in the whole disc, only notum and hinge, and only wing pouch.
(TIF)

Figure S4 The progression of pattern, in absolute time, in discs from larvae with altered developmental time and from two parental lines. The probability (represented by the size of the circle) that a disc with a particular set of gene-specific stages belongs to a given disc stage, varied with absolute developmental time (hours after third instar ecdysis (h AL3E)). (a-b) Temperature manipulations include (a) 18°C in blue and (b) 29°C in orange. (c-d) We manipulated the timing of ecdysone synthesis using (c) *P0206>PTEN* larvae (in green) and (d) *phm>InR* larvae (in purple). (e-f) Parental lines to test for the contribution of genetic background include (e) *>PTEN* in red and (e) *>InR* in pink. Developmental events are identified by m (moulting), w (wandering) and wpp (white pre-pupae).
(TIF)

Figure S5 Patterning progression of four gene products in discs from wild-type larvae reared at 18°C. The expression of Achaete (1a-1h), Senseless (2a-2h) and Dachshund (3a-3h) at 0 (1a-3a), 10 (1b-3b), 20 (1c-3c), 30 (1d-3d), 50 (1e-3e) and 70 (1f-3f) hours after third instar ecdysis (h AL3E), wandering (1g-3g) (at the average time of 96 h AL3E) and white pre-pupae (at the average time of 101 h AL3E, 1h-3h). Wingless expression is represented only for the moult to the third instar (0h AL3E, 4a). Arrows show addition or change of cells or patches of cells, and asterisks highlight changes in stripes. Under each time point is the corresponding relative developmental time (normalized to pupariation). In green under each disc is the attributed gene-specific stage. (i-k) For each time point, the size of each circle represents the proportion of discs attributed to each gene-specific stage, represented in relative developmental time: (i) Achaete (Ac) stages, (j) Senseless (Sens) stages and (k) Dachshund (Dac) stages. The differences in axis spacing between gene-specific stages scale according to developmental time at 25°C. For example, the transition from Ac stage 1 to 2 takes 5 h while the transition from Ac stage 6 to 7 takes 15 h. Wild type 18°C staged discs are represented in blue while the 25°C staged discs from our staging scheme are in black. Developmental events are identified by m (moulting), w (wandering) and wpp (white pre-pupae).
(TIF)

Figure S6 Patterning progression of four gene products in discs from wild-type larvae reared at 29°C. The expression of Achaete (1a-1h), Senseless (2a-2h) and Dachshund (3a-3h) shown at 0 (1a-3a), 10 (1b-3b), 20 (1c-3c), 30 (1d-3d), 35 (1e-3e) and 40 (1f-3f) hours after third instar ecdysis (h AL3E), wandering (1g-3g) (at the average time of 48 h AL3E) and white pre-pupae (at the average time of 52 h AL3E, 1h-3h). Wingless expression is represented only for the moult to the third instar (0h AL3E, 4a). Arrows show addition or change in the appearance of cells or patches of cells, and asterisks highlight changes in stripes. Under each time point is the corresponding relative developmental time (normalized to pupariation). In green under each disc is the attributed gene-specific stage. (i-k) For each time point, the size of each circle represents the proportion of discs attributed to each gene-specific stage in relative developmental time: (i) Achaete (Ac) stages, (j) Senseless (Sens) stages and (k) Dachshund (Dac) stages. The differences in axis spacing between gene-specific stages scale according to developmental time at 25°C. For example, the transition from Ac stage 1 to 2 takes 5 h while the transition from Ac stage 6 to 7 takes 15 h. Wild type 29°C staged discs are represented in orange while the 25°C staged discs from our staging scheme are in black. Developmental events are identified by m (moulting), w (wandering) and wpp (white pre-pupae).
(TIF)

Figure S7 Comparing expression patterns at moulting, wandering and pupariation in larvae reared at 25°C and 29°C. The expression patterns of Ac, Sens, Dac and Wg between discs from wild-type larvae reared at 25°C and reared at 29°C at the three developmental events of moulting, wandering and pupariation. Comparison of the expression of Achaete (a, e, i, l, o, r), Senseless (b, f, j, m, p, s), Dachshund (c, g, k, n, q, t) and Wingless (d, h) at the moult to the third instar (0h, a-h), wandering (i-n) and pupariation (o-t) between wild-type larvae reared at 25°C (a-d, i-k, o-q) and reared at 29°C (e-h, l-n, r-t). The corresponding disc stages are represented in the column to the right of the images. Arrows show addition or change of cells or patches of cells, and asterisks highlight changes in stripes. Scale bar is 100 µm.
(TIF)

Figure S8 Patterning progression of four gene products in discs from larvae reared with delayed ecdysone production (*P0206>PTEN*). The expression of Achaete (1a-1h), Senseless (2a-2h) and Dachshund (3a-3h) shown at 0 (1a-3a), 10 (1b-3b), 20 (1c-3c), 30 (1d-3d), 40 (1e-3e) and 60 (1f-3f) hours after third instar ecdysis (h AL3E), wandering (1g-3g) (at the average time of 73 h AL3E) and white pre-pupae (at the average time of 80 h AL3E, 1h-3h). Wingless expression is represented only for the moult to the third instar (0h AL3E, 4a). Arrows mark the addition or change of cells or patches of cells, and asterisks highlight changes in stripes. Under each time point is the corresponding relative developmental time (normalized to pupariation). In green under each disc is the attributed gene-specific stage. (i-k) For each time point, the size of each circle represents the proportion of discs attributed to each gene-specific stage in relative developmental time: (i) Achaete (Ac) stages, (j) Senseless (Sens) stages and (k) Dachshund (Dac) stages. The differences in axis spacing between gene-specific stages scale according to developmental time at 25°C. For example, the transition from Ac stage 1 to 2 takes 5 h while the transition from Ac stage 6 to 7 takes 15 h. *P0206>PTEN* staged discs are represented in green while the 25°C staged discs from our staging scheme are in black. Developmental events are identified by m (moulting), w (wandering) and wpp (white pre-pupae).
(TIF)

Figure S9 Patterning progression of four gene products in discs from larvae with accelerated ecdysone production (*phm>InR*). The expression of Achaete (1a-1f), Senseless (2a-2f) and Dachshund (3a-3f) shown at 0 (1a-3a), 10 (1b-3b), 20 (1c-3c) and 30 (1d-3d) hours after third instar ecdysis (h AL3E), wandering (1e-3e, samples from 30.5 h AL3E) and white pre-pupae (1f-3f). Wingless expression is represented only for the moult to the third instar (0h AL3E, 4a). Arrows mark the addition or change of cells or patches of cells, and asterisks highlight changes in stripes. Under each time point is the corresponding relative developmental time (normalized to pupariation). In green under each disc is the attributed gene-specific stage. (g-i) For each time point, the size of each circle represents the proportion of discs attributed to each gene-specific stage in relative developmental time: (g) Achaete (Ac) stages, (h) Senseless (Sens) stages and (i) Dachshund (Dac) stages. The differences in axis spacing between gene-specific stages scale according to developmental time at 25°C. For example, the transition from Ac stage 1 to 2 takes 5 h while the transition from Ac stage 6 to 7 takes 15 h. *phm>InR* staged discs are represented in purple while the 25°C staged discs from our staging scheme are in black. Developmental events are identified by m (moulting), w (wandering) and wpp (white pre-pupae).
(TIF)

Figure S10 Patterning progression of four gene products in discs from the parental line *>PTEN*. The expression of Achaete (1a-1f), Senseless (2a-2f) and Dachshund (3a-3f) shown at 0 (1a-3a), 10 (1b-3b), 20 (1c-3c) and 30 (1d-3d) hours after third instar ecdysis (h AL3E), wandering (at the average time of 48 h AL3E, 1e-3e) and white pre-pupae (at the average time of 53 h AL3E, 1f-3f). Wingless expression is represented only for the moult to the third instar (0h AL3E, 4a). Arrows mark the addition or change of cells or patches of cells, and asterisks highlight changes in stripes. Under each time point is the corresponding relative developmental time (normalized to pupariation). In green under each disc is the attributed gene-specific stage. (g-i) For each time point, the size of each circle represents the proportion of discs attributed to each gene-specific stage in relative developmental time: (g) Achaete (Ac) stages, (h) Senseless (Sens) stages and (i) Dachshund (Dac) stages. The differences in axis spacing between gene-specific stages scale according to developmental time at 25°C. For example, the transition from Ac stage 1 to 2 takes 5 h while the transition from Ac stage 6 to 7 takes 15 h. *>PTEN* staged discs are represented in red while the wild type 25°C staged discs from our staging scheme are in black. Developmental events are identified by m (moulting), w (wandering) and wpp (white pre-pupae).
(TIF)

Figure S11 Patterning progression of four gene products in discs from the parental line *>InR*. The expression of Achaete (1a-1f), Senseless (2a-2f) and Dachshund (3a-3f) shown at 0 (1a-3a), 10 (1b-3b), 20 (1c-3c) and 30 (1d-3d) hours after third instar ecdysis (h AL3E), wandering (at the average time of 48 h AL3E, 1e-3e) and white pre-pupae (at the average time of 51 h AL3E, 1f-3f). Wingless expression is represented only for the moult to the third instar (0h AL3E, 4a). Arrows mark the addition or change of cells or patches of cells, and asterisks highlight changes in stripes. Under each time point is the corresponding relative developmental time (normalized to pupariation). In green under each disc is the attributed gene-specific stage. (g-i) For each time point, the size of each circle represents the proportion of discs attributed to each gene-specific stage in relative developmental time: (g) Achaete (Ac) stages, (h) Senseless (Sens) stages and (i) Dachshund (Dac) stages. The differences in axis spacing between gene-specific stages scale according to developmental time at 25°C. For example, the transition from Ac stage 1 to 2 takes 5 h while the transition from Ac stage 6 to 7 takes 15 h. *>InR* staged discs are represented in pink while the wild type 25°C staged discs from our staging scheme are in black. Developmental events are identified by m (moulting), w (wandering) and wpp (white pre-pupae).
(TIF)

Figure S12 The progression of pattern, in relative and absolute time, in discs from larvae with altered timing of ecdysis synthesis and respective parental lines. Probability (represented by the size of the circle) that a disc with a particular set of gene-specific stages belongs to a given disc stage varied by relative (normalized to pupariation)(a, b) or absolute developmental time (hours after third instar ecdysis (h AL3E))(c, d). Manipulations of the timing of ecdysis synthesis: (a, c) disc stages attributed to discs from *P0206>PTEN* larvae are shown in green and disc stages attributed to discs from *>PTEN* larvae are in red; (b, d) disc stages attributed to discs from *phm>InR* larvae are shown in purple and disc stages attributed to discs from *>InR* larvae are in pink. Developmental events are identified by m (moulting), w (wandering) and wpp (white pre-pupae).
(TIF)

Table S1 Number of discs dissected for the wild type at 25°C for each gene product at all time points, used to devise the staging scheme. The asterisk represents discs that were simultaneously scored for both Achaete (Ac) and Senseless (Sens).
(TIF)

Table S2 Number of discs dissected for all treatments/genotypes (except for wild type at 25°C) and for each gene product at all time points. The asterisk represents discs that were simultaneously scored for both Achaete (Ac) and Senseless (Sens).
(TIF)

Table S3 List of antibodies used in the immunocytochemistry protocol. Mouse anti-Achaete was used in combination with guinea pig anti-Senseless [38].
(TIF)
