# Supplementary figures and images for: Coordination of Wing and Whole-Body Development at Developmental Milestones Ensures Robustness against Environmental and Physiological Perturbations
Source: PLoS Genet. 2014 Jun 19;10(6):e1004408. doi: 10.1371/journal.pgen.1004408 (PMC4063698; doi:10.1371/journal.pgen.1004408)

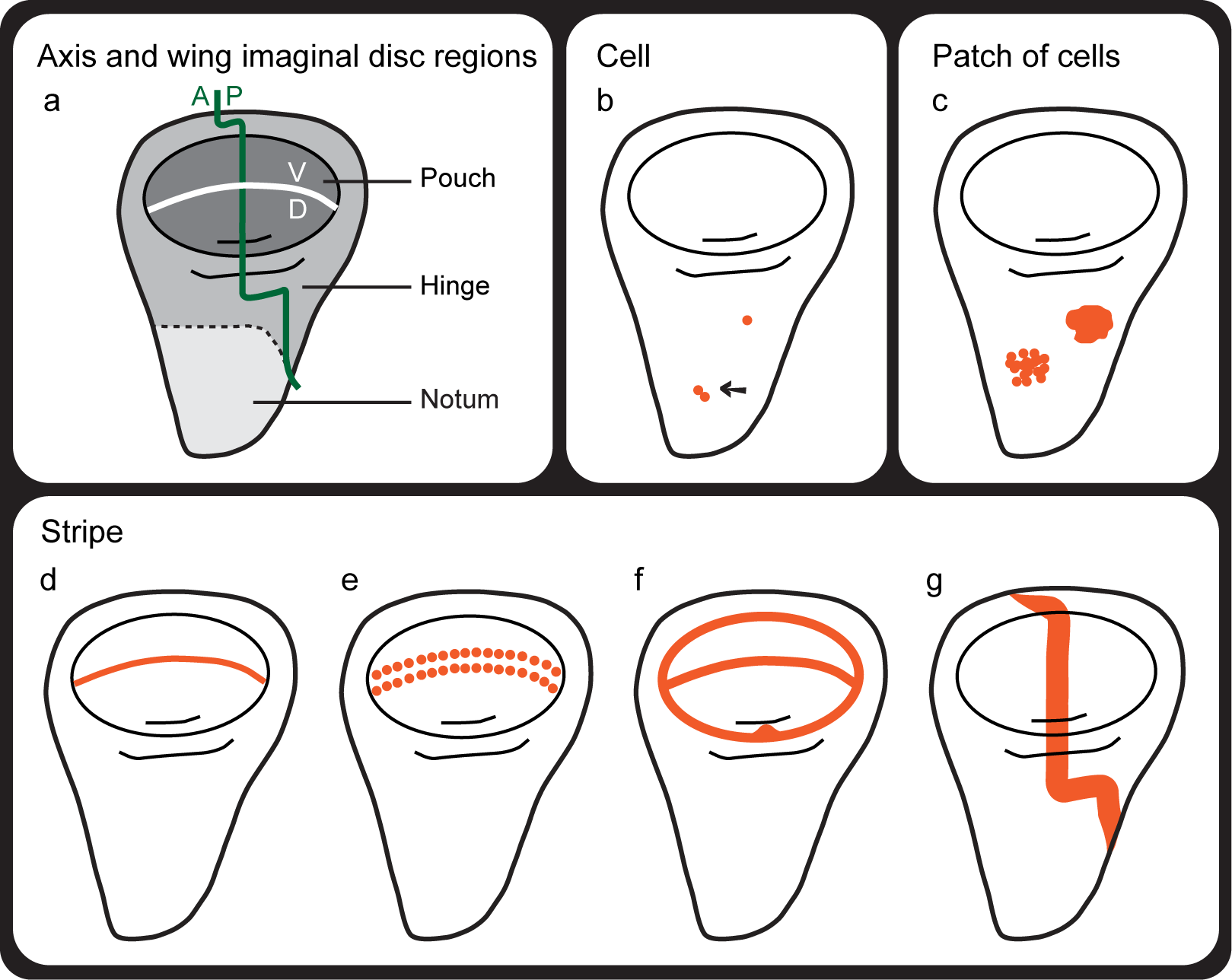

Supplement: Figure S1 — Definitions of patterning elements used to characterize stages in the staging scheme. (a) The third instar wing disc is already subdivided into domains that will form the wing pouch, wing hinge and notum of the adult fly. It has an anterior (A) and posterior (P) axis and dorsal (D) and ventral (V) domains. (b) The element cell was defined by one round dot of expression, which corresponded to the refinement of expression to a sensory organ precursor (SOP). For later time points, SOPs divide giving rise to two sister SOPs, referred as doublets (arrow). (c) Patch of cells refers to a region of pattern that resembles a cluster of cells, either clearly delimited or diffuse. (d-g) Stripe corresponds to one or more line of cells (more or less defined) (d, e), that can be parallel (double stripe) or perpendicular (forming a cross) to each other, located in the developing wing pouch along the dorsal-ventral axis. Stripes also appear as lines restricted to the anterior side of the wing pouch or along the dorsal-ventral boundary with a surrounding ring (stripe with ring, f), or along the anterior-posterior axis (g). The stripes correspond to lines of SOPs along the wing margin (e), or lines of positional information regarding wing disc boundaries (d). Different compositions of these elements describe all observed patterns for each gene product through development. (TIF) [file pgen.1004408.s001.tif]

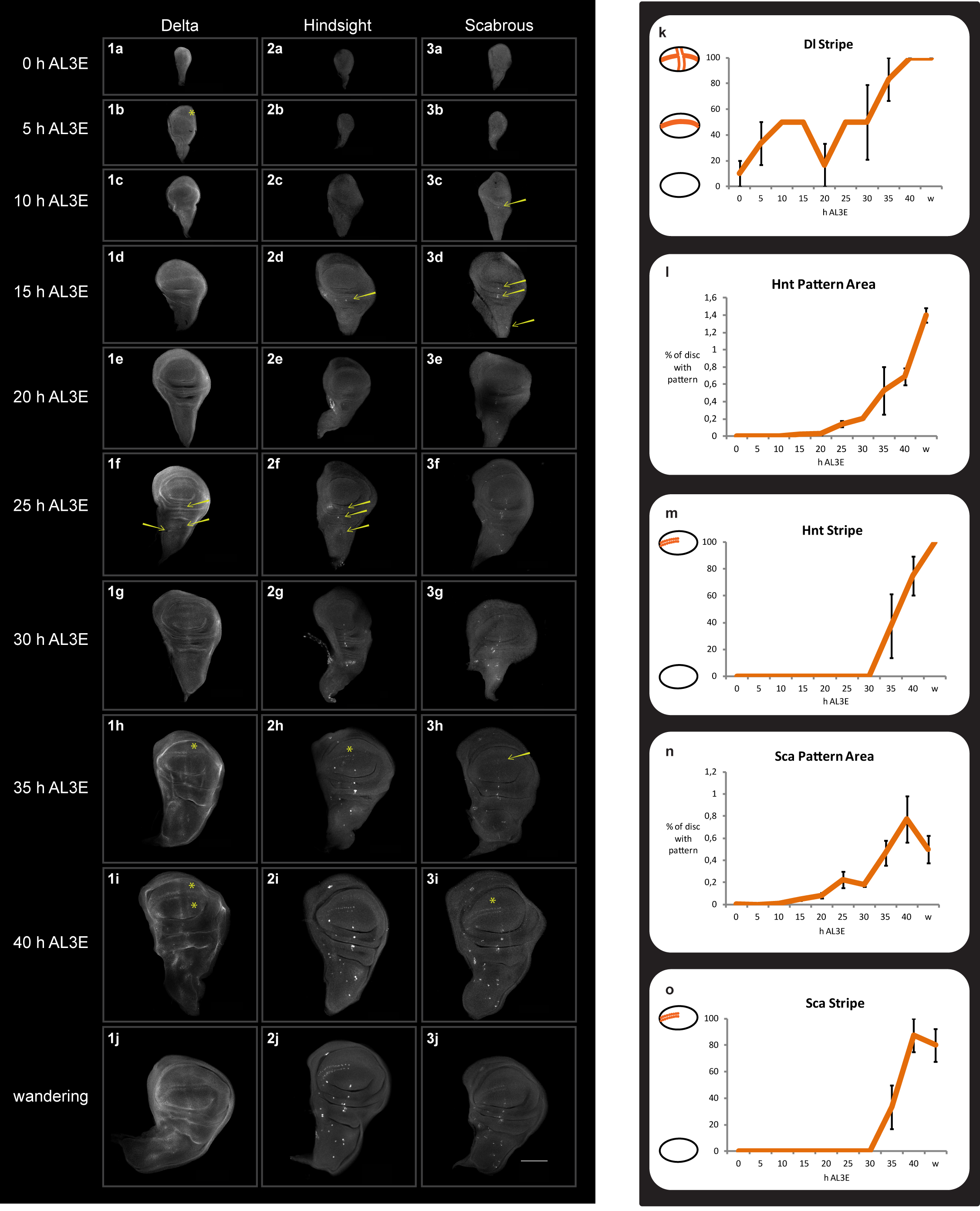

Supplement: Figure S2 — Patterning progression of three of the eleven gene products initially assessed but not included in the staging scheme. Dynamic expression of Delta (1a-1j), Hindsight (2a-2j) and Scabrous (3a-3j) at 0 (1a-3a), 5 (1b-3b), 10 (1c-3c), 15 (1d-3d), 20 (1e-3e), 25 (1f-3f), 30 (1g-3g), 35 (1h-3h), and 40 (1i-3i) hours after third instar ecdysis (h AL3E) and wandering (1j-3j). Arrows show addition or change of cells or patches of cells, and asterisks highlight changes in stripes. (1f) Arrows highlight Delta expression mainly in the hinge and notum. Hindsight undergoes four transitions adding new elements at 15, 25 and 35 h AL3E. Lastly, Scabrous undergoes four transitions adding new elements at 10, 15 and 40 h AL3E. (3h) shows Scabrous expression in the centre of the wing pouch, before it refines to a stripe. (Scale bar 100 µm). (k-o) Quantitative measures of the relative amount of expression normalized to disc size of the different elements observed. (k) Delta expression pattern represented by the progression of the stripe. (l-m) Hindsight expression pattern decomposed into (l) pattern area and (m) progression of the stripe. (n-o) Scabrous expression pattern decomposed into (n) pattern area and (o) progression of the stripe. Delta undergoes four transitions in its pattern, adding new elements at 5, 35 and 40 h AL3E. (TIF) [file pgen.1004408.s002.tif]

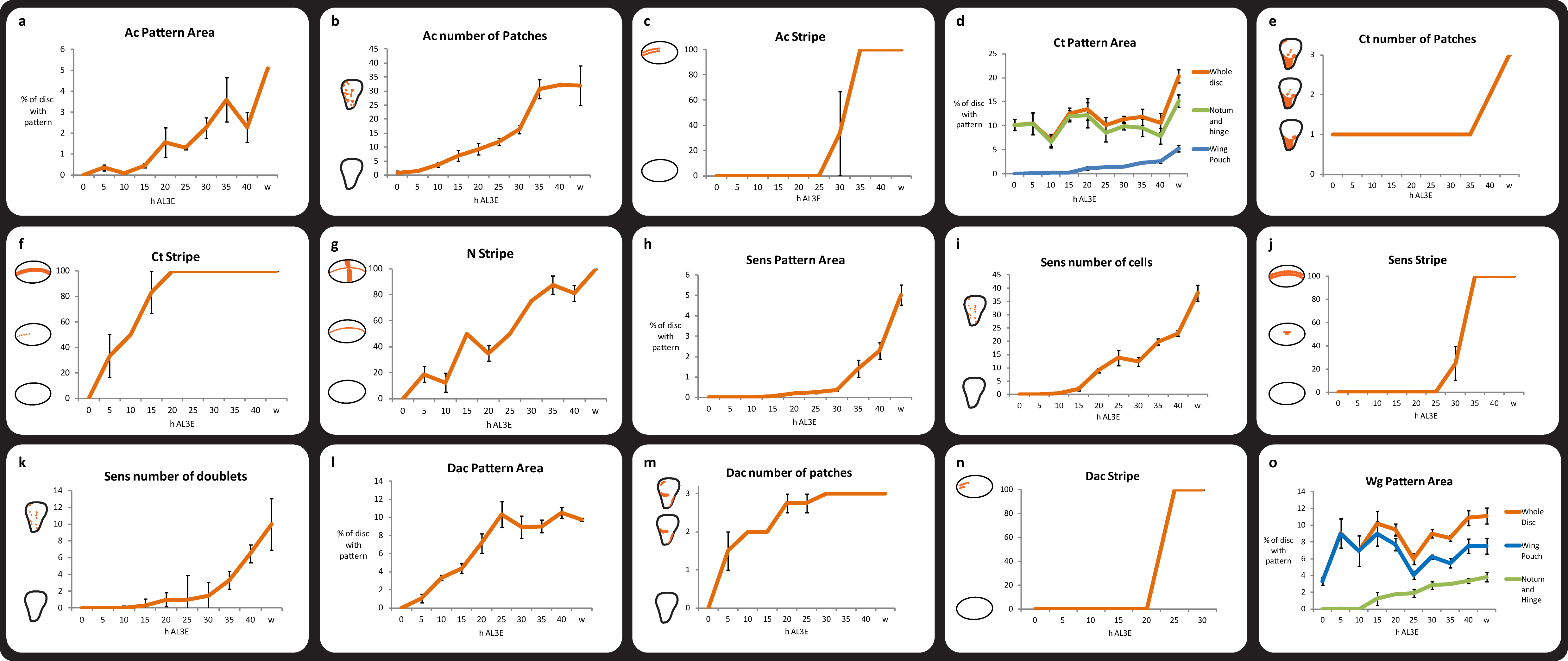

Supplement: Figure S3 — Quantitative measures of the relative amount of expression normalized to disc size and of the different elements observed for six of the eleven gene products. (a-c) Achaete expression pattern decomposed into (a) pattern area, (b) number of patches of cells and (c) progression of the stripe. (d-f) Cut pattern broken down into (d) pattern area (whole disc, only notum and hinge, and only wing pouch), (e) number of patches of cells and (f) progression of the stripe. (g) Notch expression pattern represented by the stripe progression. (h-k) Senseless expression pattern decomposed into (h) pattern area, (i) number of SOPs, (j) progression of the stripe and (k) number of doublets. (l-n) Dachshund expression pattern in terms of (l) pattern area, (m) number of patches of cells and (n) progression of the stripe. (o) Wingless pattern area in the whole disc, only notum and hinge, and only wing pouch. (TIF) [file pgen.1004408.s003.tif]

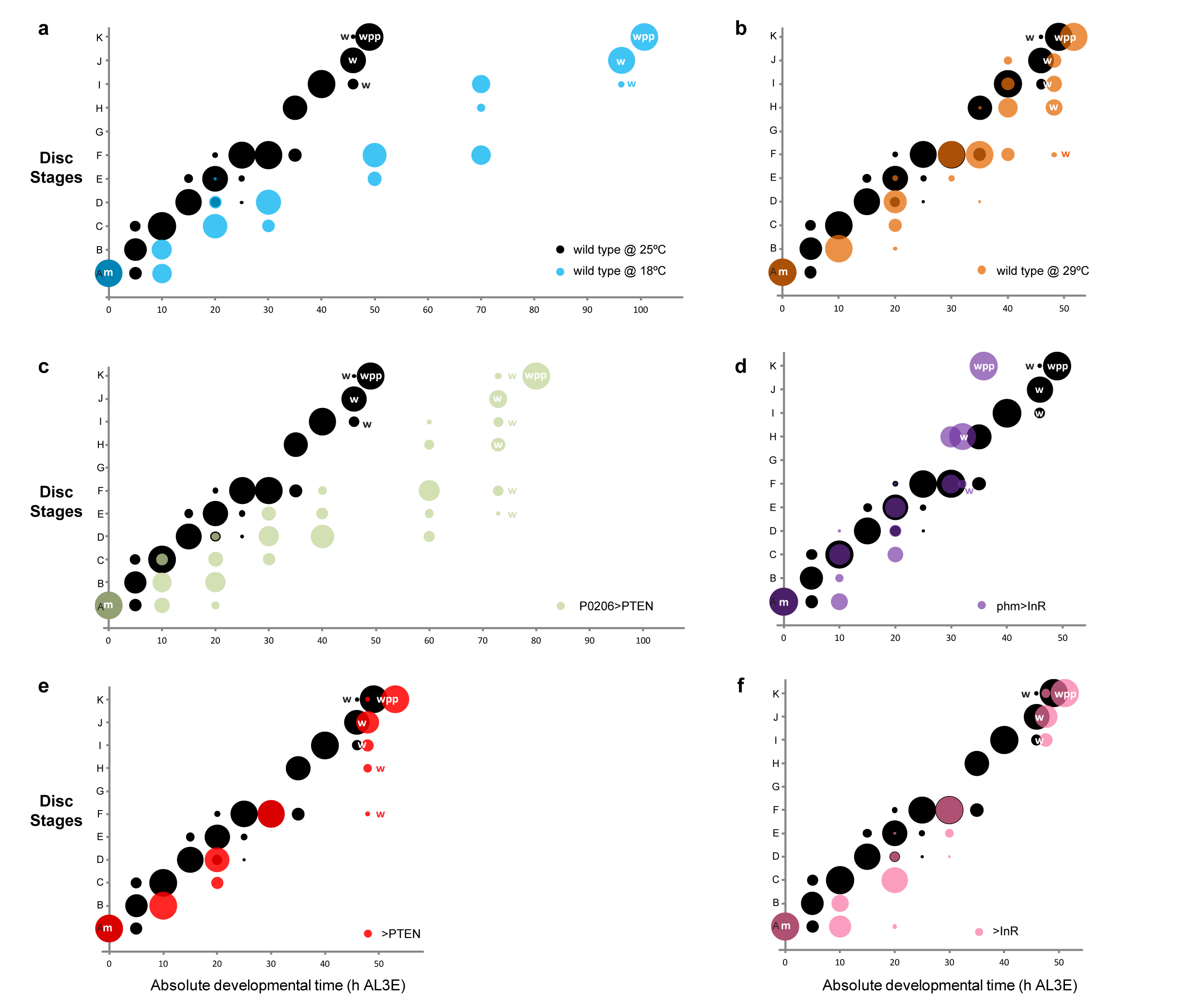

Supplement: Figure S4 — The progression of pattern, in absolute time, in discs from larvae with altered developmental time and from two parental lines. The probability (represented by the size of the circle) that a disc with a particular set of gene-specific stages belongs to a given disc stage, varied with absolute developmental time (hours after third instar ecdysis (h AL3E)). (a-b) Temperature manipulations include (a) 18°C in blue and (b) 29°C in orange. (c-d) We manipulated the timing of ecdysone synthesis using (c) P0206>PTEN larvae (in green) and (d) phm>InR larvae (in purple). (e-f) Parental lines to test for the contribution of genetic background include (e) >PTEN in red and (e) >InR in pink. Developmental events are identified by m (moulting), w (wandering) and wpp (white pre-pupae). (TIF) [file pgen.1004408.s004.tif]

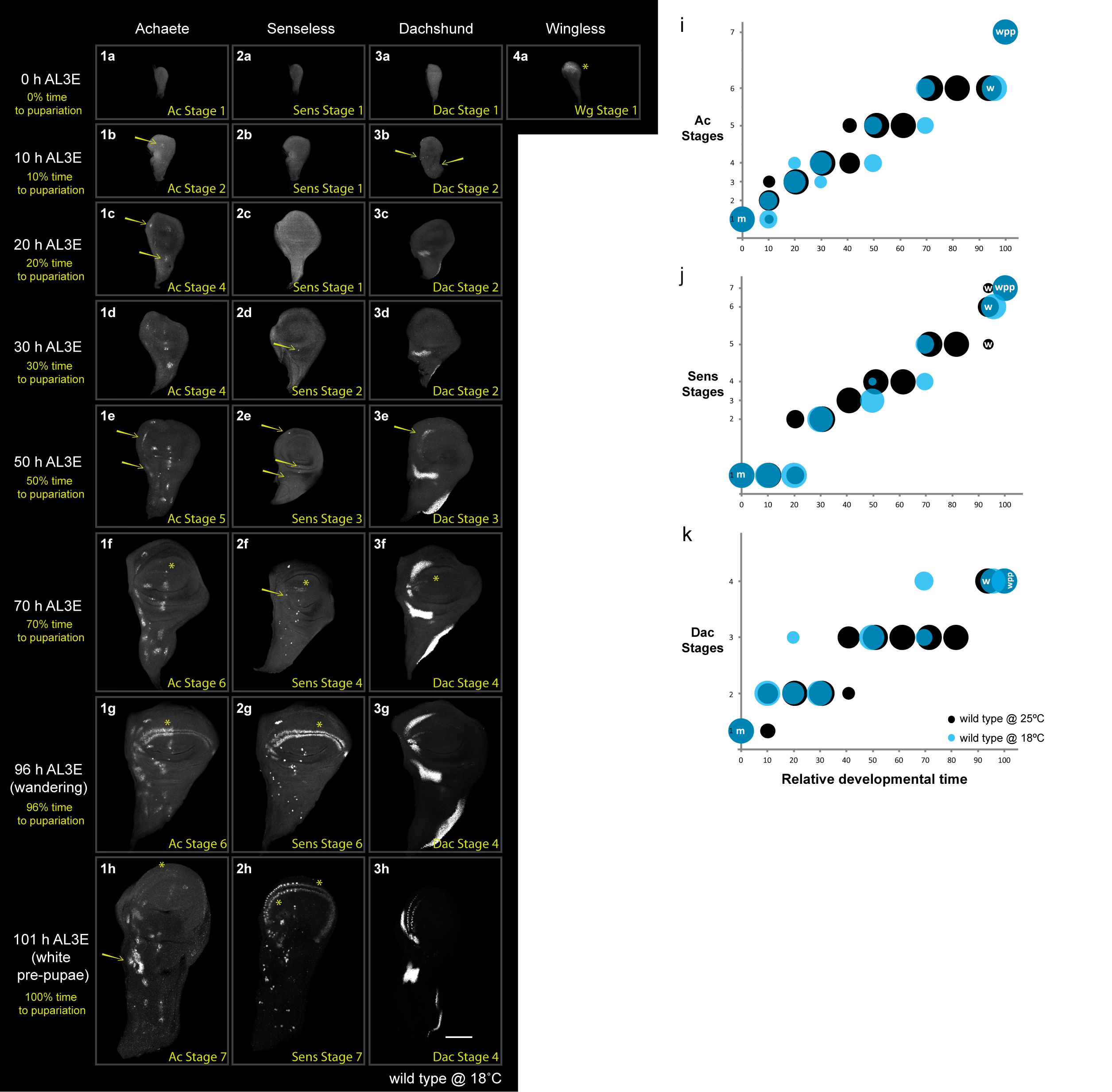

Supplement: Figure S5 — Patterning progression of four gene products in discs from wild-type larvae reared at 18°C. The expression of Achaete (1a-1h), Senseless (2a-2h) and Dachshund (3a-3h) at 0 (1a-3a), 10 (1b-3b), 20 (1c-3c), 30 (1d-3d), 50 (1e-3e) and 70 (1f-3f) hours after third instar ecdysis (h AL3E), wandering (1g-3g) (at the average time of 96 h AL3E) and white pre-pupae (at the average time of 101 h AL3E, 1h-3h). Wingless expression is represented only for the moult to the third instar (0h AL3E, 4a). Arrows show addition or change of cells or patches of cells, and asterisks highlight changes in stripes. Under each time point is the corresponding relative developmental time (normalized to pupariation). In green under each disc is the attributed gene-specific stage. (i-k) For each time point, the size of each circle represents the proportion of discs attributed to each gene-specific stage, represented in relative developmental time: (i) Achaete (Ac) stages, (j) Senseless (Sens) stages and (k) Dachshund (Dac) stages. The differences in axis spacing between gene-specific stages scale according to developmental time at 25°C. For example, the transition from Ac stage 1 to 2 takes 5 h while the transition from Ac stage 6 to 7 takes 15 h. Wild type 18°C staged discs are represented in blue while the 25°C staged discs from our staging scheme are in black. Developmental events are identified by m (moulting), w (wandering) and wpp (white pre-pupae). (TIF) [file pgen.1004408.s005.tif]

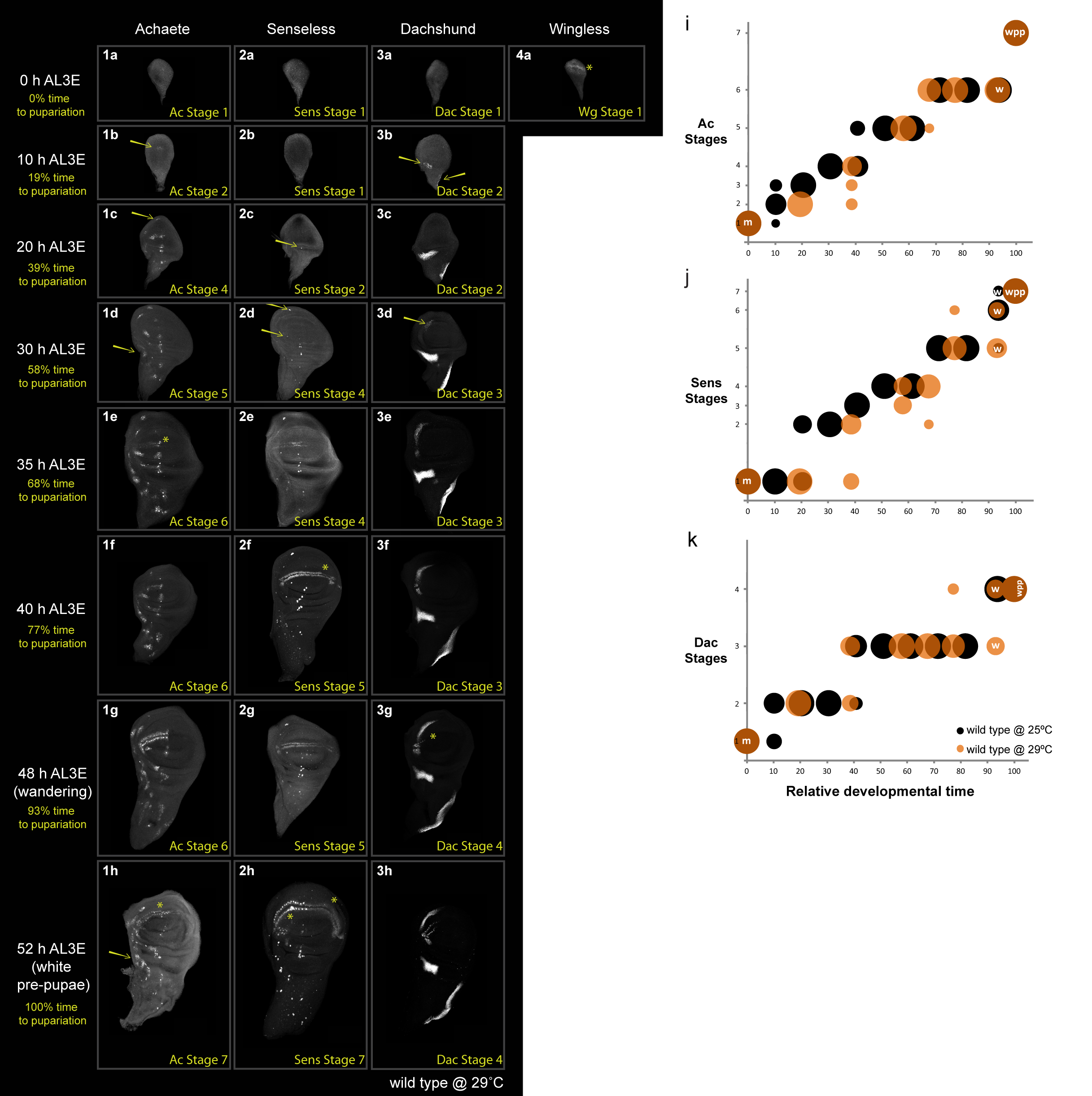

Supplement: Figure S6 — Patterning progression of four gene products in discs from wild-type larvae reared at 29°C. The expression of Achaete (1a-1h), Senseless (2a-2h) and Dachshund (3a-3h) shown at 0 (1a-3a), 10 (1b-3b), 20 (1c-3c), 30 (1d-3d), 35 (1e-3e) and 40 (1f-3f) hours after third instar ecdysis (h AL3E), wandering (1g-3g) (at the average time of 48 h AL3E) and white pre-pupae (at the average time of 52 h AL3E, 1h-3h). Wingless expression is represented only for the moult to the third instar (0h AL3E, 4a). Arrows show addition or change in the appearance of cells or patches of cells, and asterisks highlight changes in stripes. Under each time point is the corresponding relative developmental time (normalized to pupariation). In green under each disc is the attributed gene-specific stage. (i-k) For each time point, the size of each circle represents the proportion of discs attributed to each gene-specific stage in relative developmental time: (i) Achaete (Ac) stages, (j) Senseless (Sens) stages and (k) Dachshund (Dac) stages. The differences in axis spacing between gene-specific stages scale according to developmental time at 25°C. For example, the transition from Ac stage 1 to 2 takes 5 h while the transition from Ac stage 6 to 7 takes 15 h. Wild type 29°C staged discs are represented in orange while the 25°C staged discs from our staging scheme are in black. Developmental events are identified by m (moulting), w (wandering) and wpp (white pre-pupae). (TIF) [file pgen.1004408.s006.tif]

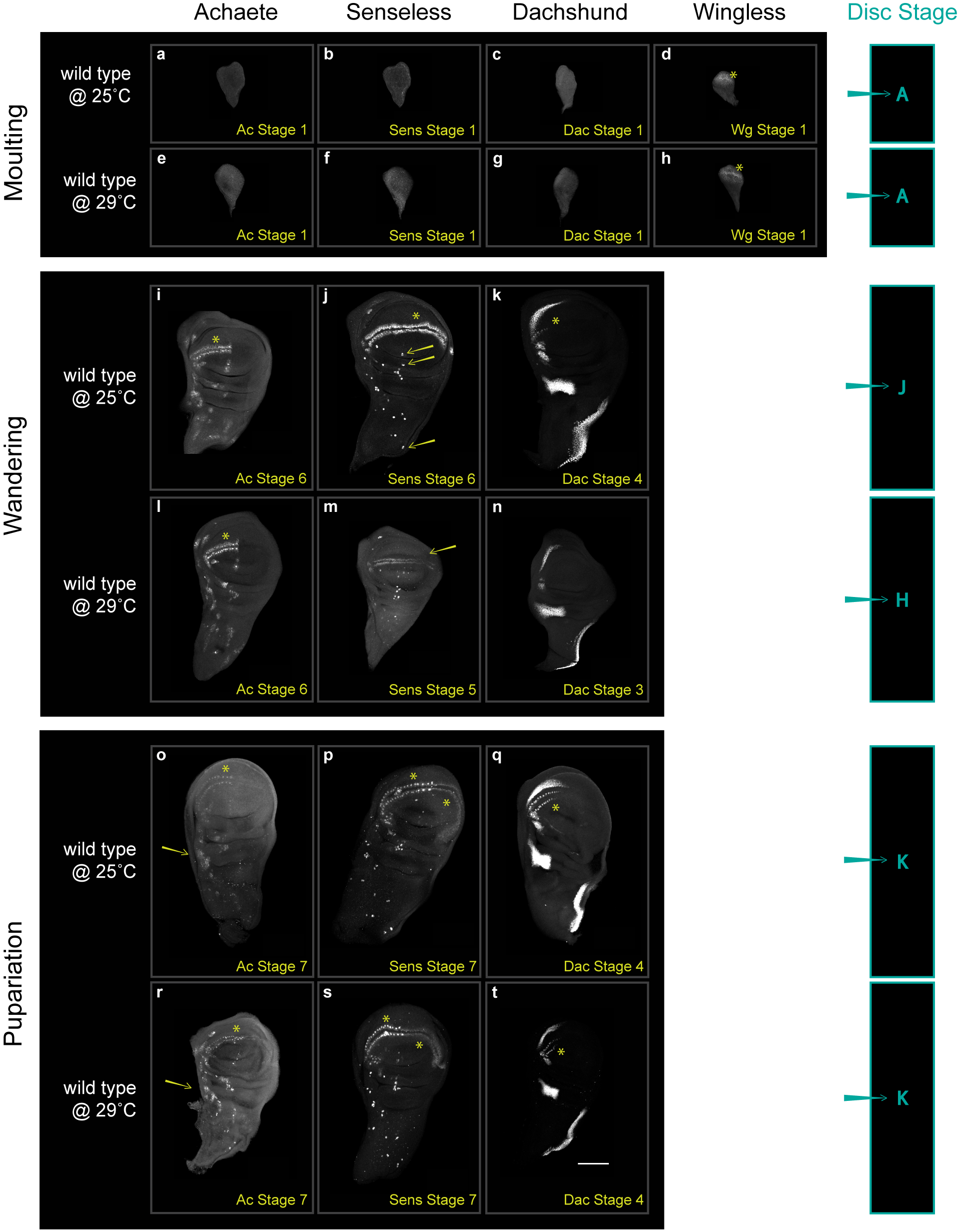

Supplement: Figure S7 — Comparing expression patterns at moulting, wandering and pupariation in larvae reared at 25°C and 29°C. The expression patterns of Ac, Sens, Dac and Wg between discs from wild-type larvae reared at 25°C and reared at 29°C at the three developmental events of moulting, wandering and pupariation. Comparison of the expression of Achaete (a, e, i, l, o, r), Senseless (b, f, j, m, p, s), Dachshund (c, g, k, n, q, t) and Wingless (d, h) at the moult to the third instar (0h, a-h), wandering (i-n) and pupariation (o-t) between wild-type larvae reared at 25°C (a-d, i-k, o-q) and reared at 29°C (e-h, l-n, r-t). The corresponding disc stages are represented in the column to the right of the images. Arrows show addition or change of cells or patches of cells, and asterisks highlight changes in stripes. Scale bar is 100 µm. (TIF) [file pgen.1004408.s007.tif]

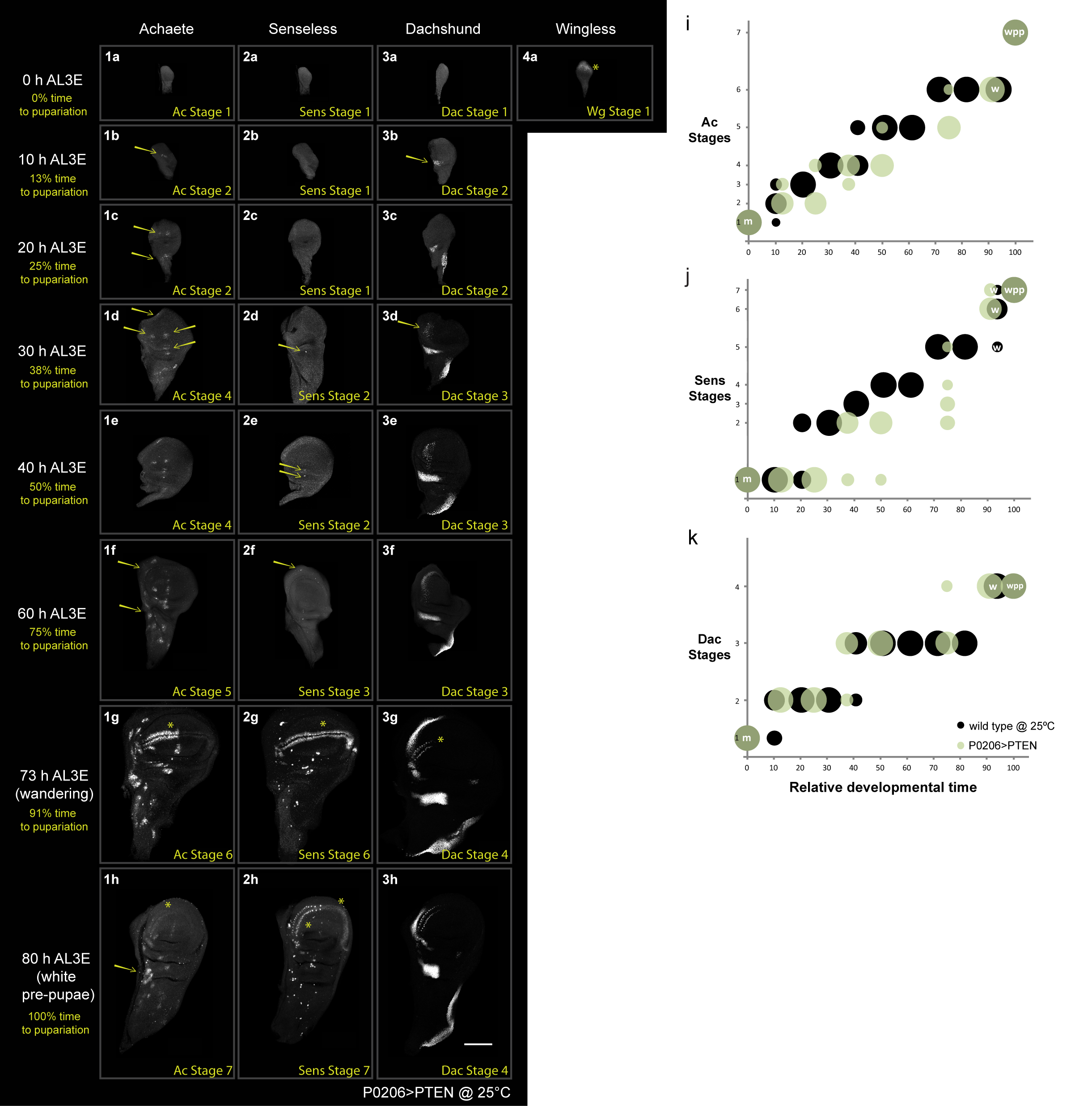

Supplement: Figure S8 — Patterning progression of four gene products in discs from larvae reared with delayed ecdysone production (P0206>PTEN). The expression of Achaete (1a-1h), Senseless (2a-2h) and Dachshund (3a-3h) shown at 0 (1a-3a), 10 (1b-3b), 20 (1c-3c), 30 (1d-3d), 40 (1e-3e) and 60 (1f-3f) hours after third instar ecdysis (h AL3E), wandering (1g-3g) (at the average time of 73 h AL3E) and white pre-pupae (at the average time of 80 h AL3E, 1h-3h). Wingless expression is represented only for the moult to the third instar (0h AL3E, 4a). Arrows mark the addition or change of cells or patches of cells, and asterisks highlight changes in stripes. Under each time point is the corresponding relative developmental time (normalized to pupariation). In green under each disc is the attributed gene-specific stage. (i-k) For each time point, the size of each circle represents the proportion of discs attributed to each gene-specific stage in relative developmental time: (i) Achaete (Ac) stages, (j) Senseless (Sens) stages and (k) Dachshund (Dac) stages. The differences in axis spacing between gene-specific stages scale according to developmental time at 25°C. For example, the transition from Ac stage 1 to 2 takes 5 h while the transition from Ac stage 6 to 7 takes 15 h. P0206>PTEN staged discs are represented in green while the 25°C staged discs from our staging scheme are in black. Developmental events are identified by m (moulting), w (wandering) and wpp (white pre-pupae). (TIF) [file pgen.1004408.s008.tif]

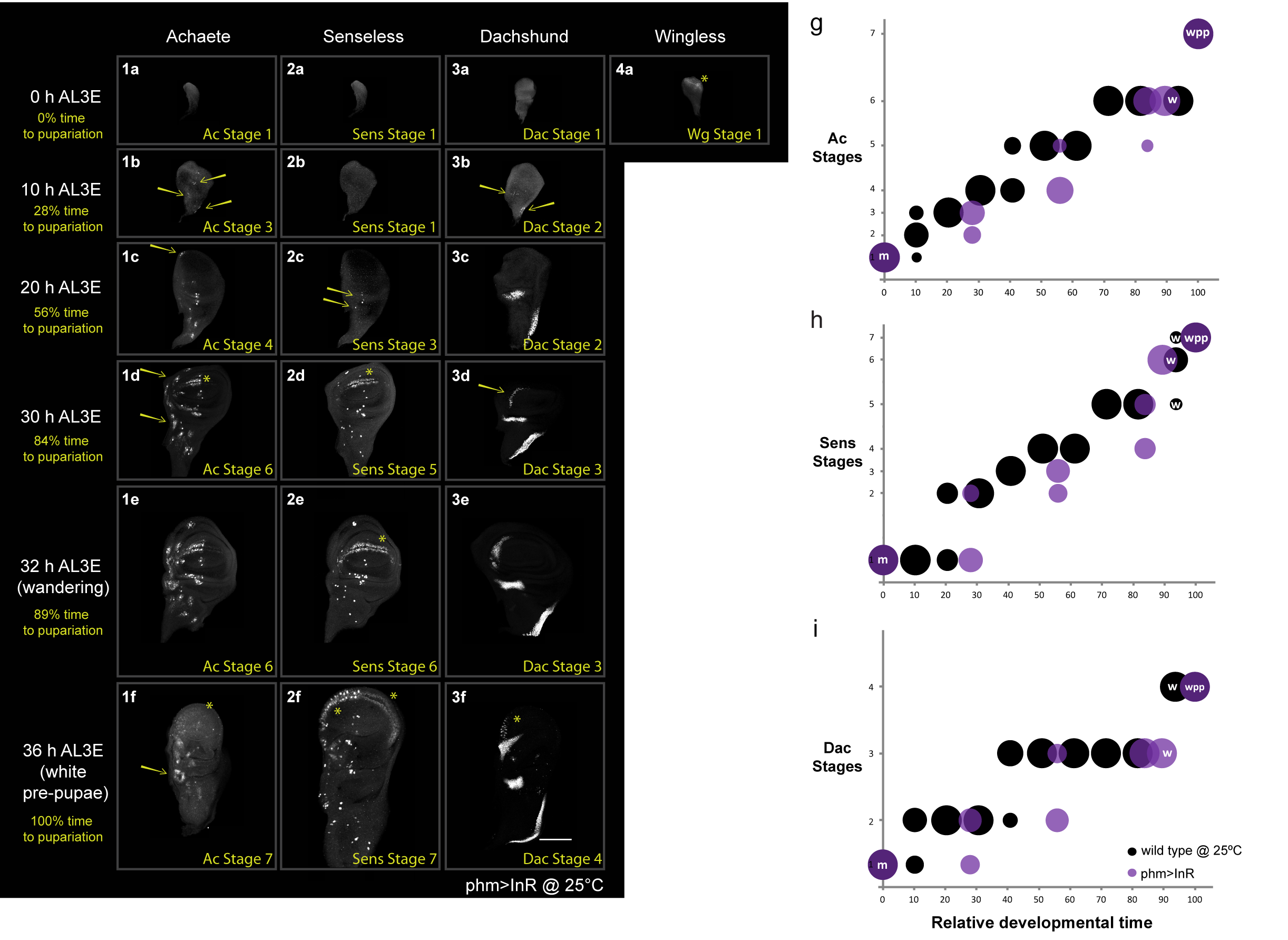

Supplement: Figure S9 — Patterning progression of four gene products in discs from larvae with accelerated ecdysone production (phm>InR). The expression of Achaete (1a-1f), Senseless (2a-2f) and Dachshund (3a-3f) shown at 0 (1a-3a), 10 (1b-3b), 20 (1c-3c) and 30 (1d-3d) hours after third instar ecdysis (h AL3E), wandering (1e-3e, samples from 30.5 h AL3E) and white pre-pupae (1f-3f). Wingless expression is represented only for the moult to the third instar (0h AL3E, 4a). Arrows mark the addition or change of cells or patches of cells, and asterisks highlight changes in stripes. Under each time point is the corresponding relative developmental time (normalized to pupariation). In green under each disc is the attributed gene-specific stage. (g-i) For each time point, the size of each circle represents the proportion of discs attributed to each gene-specific stage in relative developmental time: (g) Achaete (Ac) stages, (h) Senseless (Sens) stages and (i) Dachshund (Dac) stages. The differences in axis spacing between gene-specific stages scale according to developmental time at 25°C. For example, the transition from Ac stage 1 to 2 takes 5 h while the transition from Ac stage 6 to 7 takes 15 h. phm>InR staged discs are represented in purple while the 25°C staged discs from our staging scheme are in black. Developmental events are identified by m (moulting), w (wandering) and wpp (white pre-pupae). (TIF) [file pgen.1004408.s009.tif]

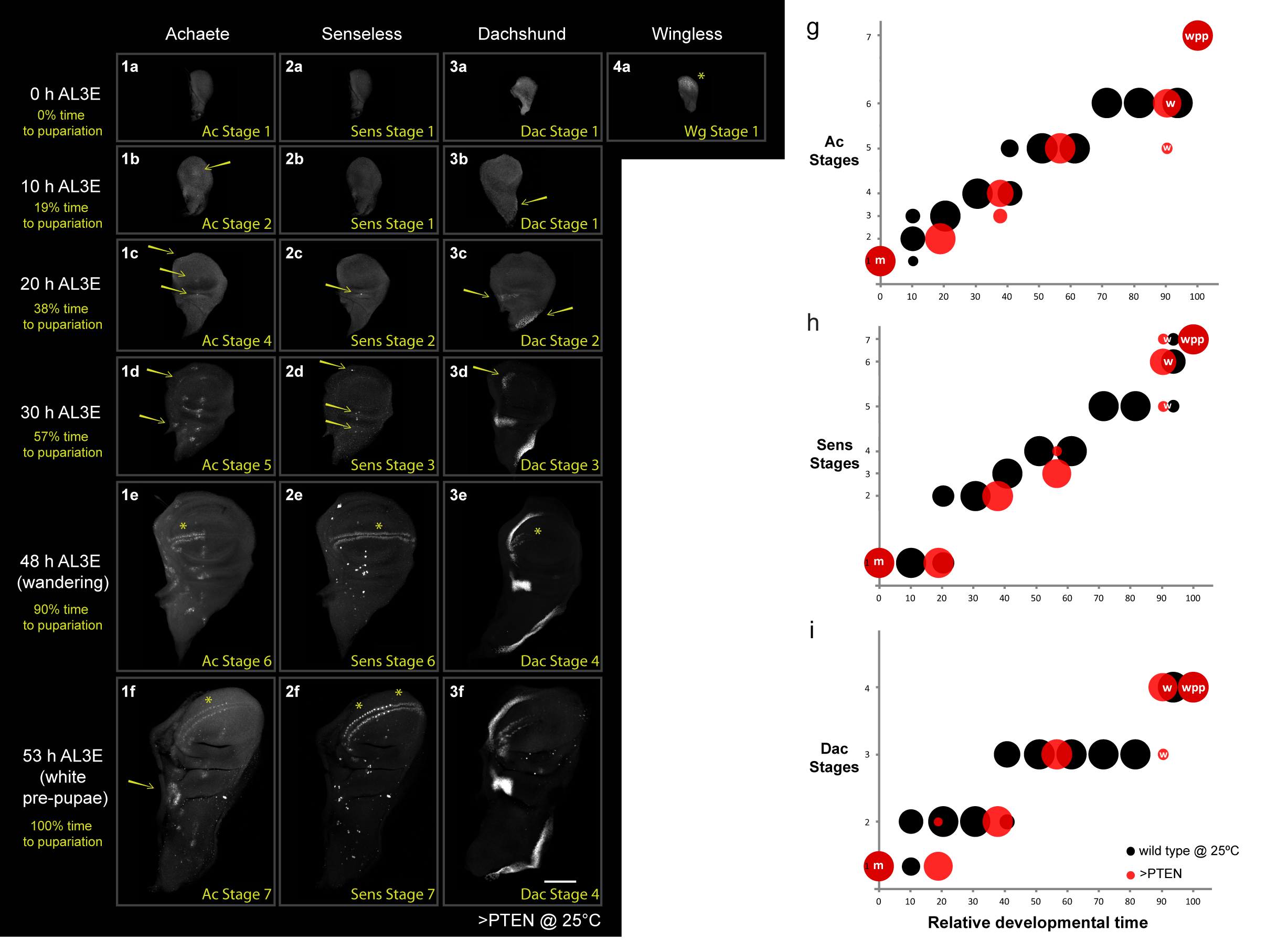

Supplement: Figure S10 — Patterning progression of four gene products in discs from the parental line >PTEN. The expression of Achaete (1a-1f), Senseless (2a-2f) and Dachshund (3a-3f) shown at 0 (1a-3a), 10 (1b-3b), 20 (1c-3c) and 30 (1d-3d) hours after third instar ecdysis (h AL3E), wandering (at the average time of 48 h AL3E, 1e-3e) and white pre-pupae (at the average time of 53 h AL3E, 1f-3f). Wingless expression is represented only for the moult to the third instar (0h AL3E, 4a). Arrows mark the addition or change of cells or patches of cells, and asterisks highlight changes in stripes. Under each time point is the corresponding relative developmental time (normalized to pupariation). In green under each disc is the attributed gene-specific stage. (g-i) For each time point, the size of each circle represents the proportion of discs attributed to each gene-specific stage in relative developmental time: (g) Achaete (Ac) stages, (h) Senseless (Sens) stages and (i) Dachshund (Dac) stages. The differences in axis spacing between gene-specific stages scale according to developmental time at 25°C. For example, the transition from Ac stage 1 to 2 takes 5 h while the transition from Ac stage 6 to 7 takes 15 h. >PTEN staged discs are represented in red while the wild type 25°C staged discs from our staging scheme are in black. Developmental events are identified by m (moulting), w (wandering) and wpp (white pre-pupae). (TIF) [file pgen.1004408.s010.tif]

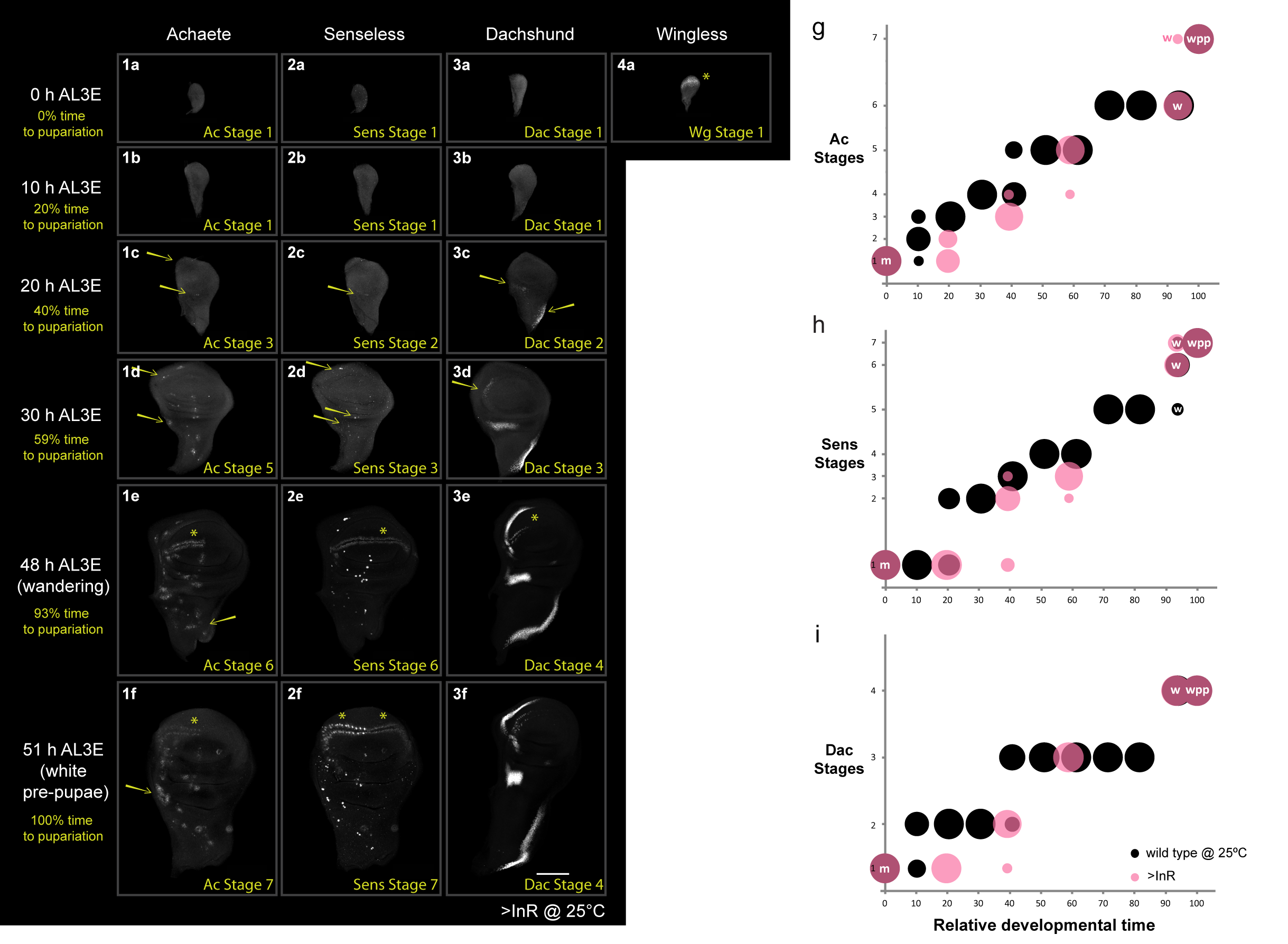

Supplement: Figure S11 — Patterning progression of four gene products in discs from the parental line >InR. The expression of Achaete (1a-1f), Senseless (2a-2f) and Dachshund (3a-3f) shown at 0 (1a-3a), 10 (1b-3b), 20 (1c-3c) and 30 (1d-3d) hours after third instar ecdysis (h AL3E), wandering (at the average time of 48 h AL3E, 1e-3e) and white pre-pupae (at the average time of 51 h AL3E, 1f-3f). Wingless expression is represented only for the moult to the third instar (0h AL3E, 4a). Arrows mark the addition or change of cells or patches of cells, and asterisks highlight changes in stripes. Under each time point is the corresponding relative developmental time (normalized to pupariation). In green under each disc is the attributed gene-specific stage. (g-i) For each time point, the size of each circle represents the proportion of discs attributed to each gene-specific stage in relative developmental time: (g) Achaete (Ac) stages, (h) Senseless (Sens) stages and (i) Dachshund (Dac) stages. The differences in axis spacing between gene-specific stages scale according to developmental time at 25°C. For example, the transition from Ac stage 1 to 2 takes 5 h while the transition from Ac stage 6 to 7 takes 15 h. >InR staged discs are represented in pink while the wild type 25°C staged discs from our staging scheme are in black. Developmental events are identified by m (moulting), w (wandering) and wpp (white pre-pupae). (TIF) [file pgen.1004408.s011.tif]

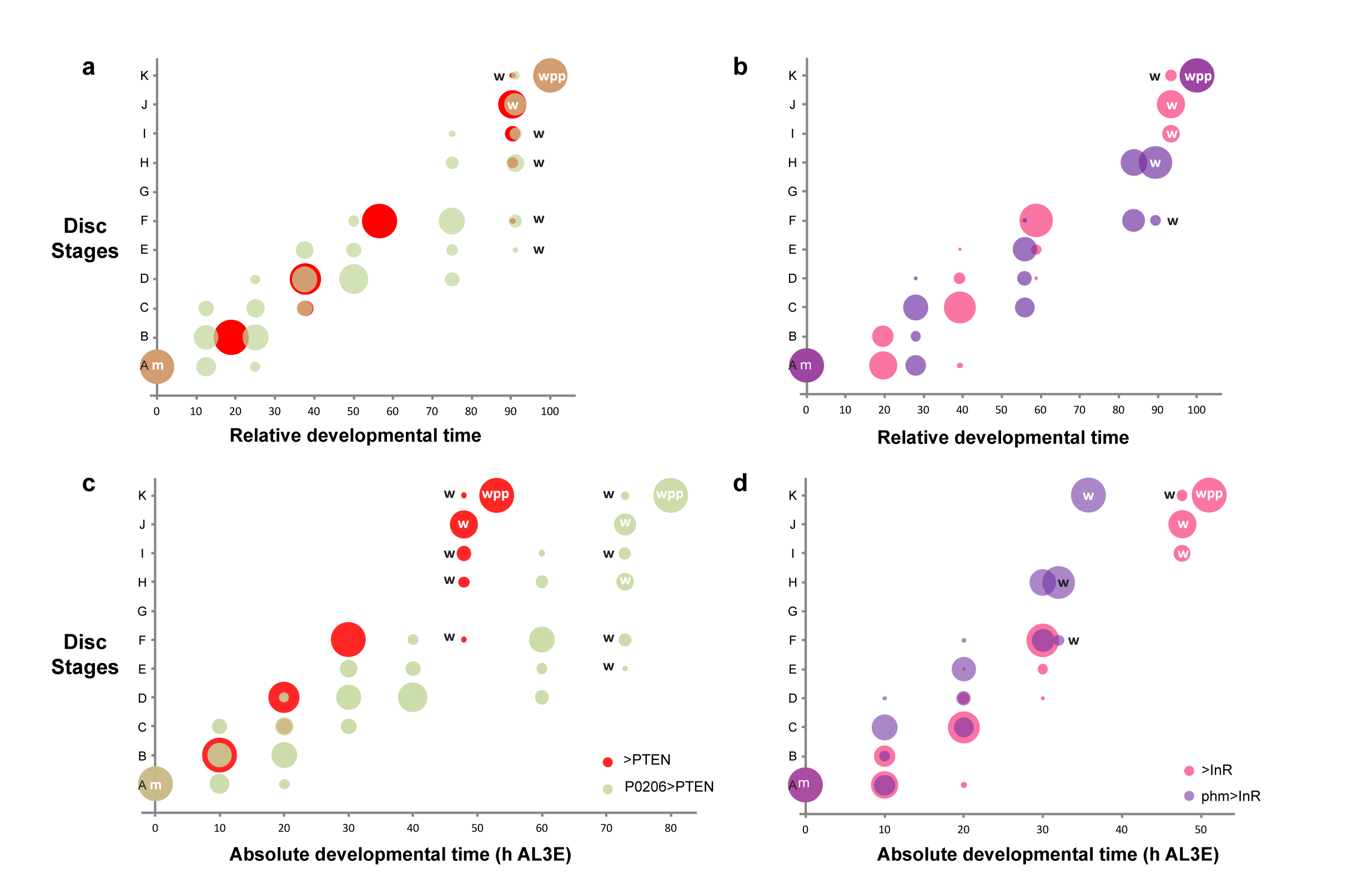

Supplement: Figure S12 — The progression of pattern, in relative and absolute time, in discs from larvae with altered timing of ecdysis synthesis and respective parental lines. Probability (represented by the size of the circle) that a disc with a particular set of gene-specific stages belongs to a given disc stage varied by relative (normalized to pupariation)(a, b) or absolute developmental time (hours after third instar ecdysis (h AL3E))(c, d). Manipulations of the timing of ecdysis synthesis: (a, c) disc stages attributed to discs from P0206>PTEN larvae are shown in green and disc stages attributed to discs from >PTEN larvae are in red; (b, d) disc stages attributed to discs from phm>InR larvae are shown in purple and disc stages attributed to discs from >InR larvae are in pink. Developmental events are identified by m (moulting), w (wandering) and wpp (white pre-pupae). (TIF) [file pgen.1004408.s012.tif]

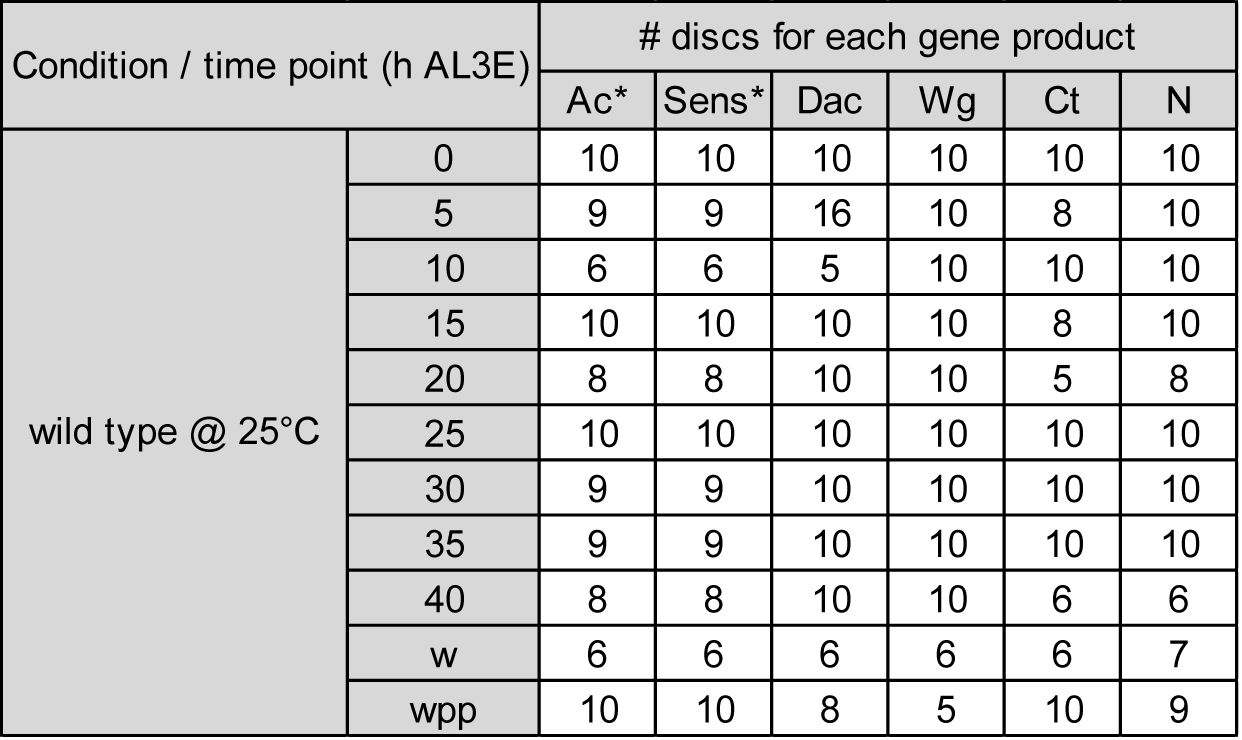

Supplement: Table S1 — Number of discs dissected for the wild type at 25°C for each gene product at all time points, used to devise the staging scheme. The asterisk represents discs that were simultaneously scored for both Achaete (Ac) and Senseless (Sens). (TIF) [file pgen.1004408.s013.tif]

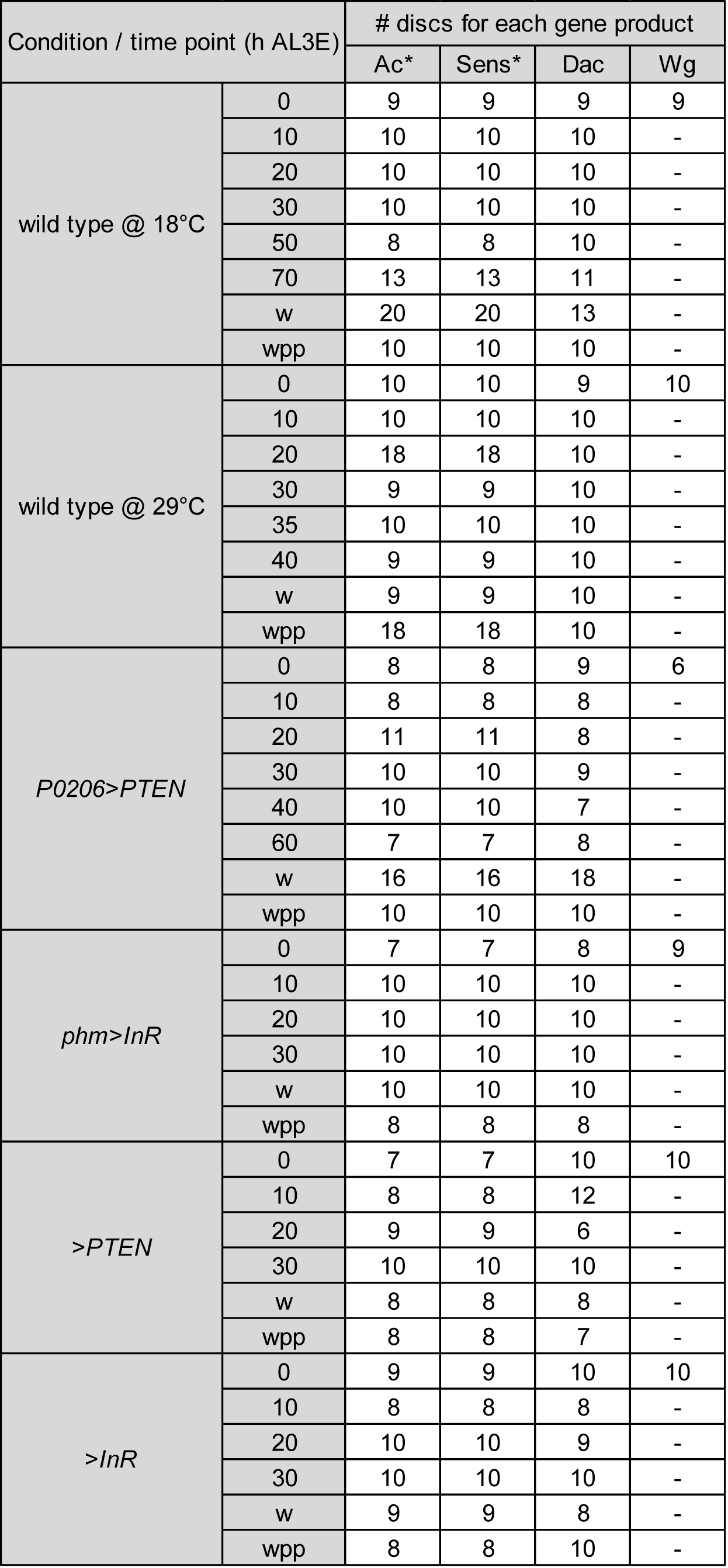

Supplement: Table S2 — Number of discs dissected for all treatments/genotypes (except for wild type at 25°C) and for each gene product at all time points. The asterisk represents discs that were simultaneously scored for both Achaete (Ac) and Senseless (Sens). (TIF) [file pgen.1004408.s014.tif]

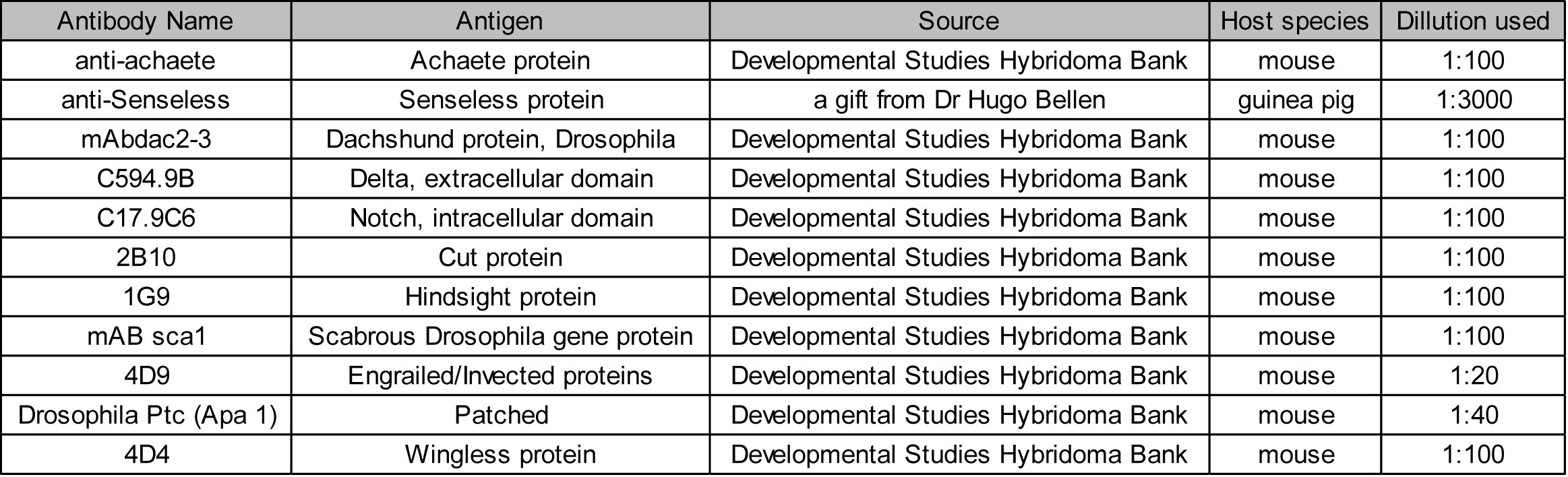

Supplement: Table S3 — List of antibodies used in the immunocytochemistry protocol. Mouse anti-Achaete was used in combination with guinea pig anti-Senseless [38]. (TIF) [file pgen.1004408.s015.tif]
